# Patient involvement in the development of patient‐reported outcome measures: a scoping review

**DOI:** 10.1111/hex.12442

**Published:** 2016-02-18

**Authors:** Bianca Wiering, Dolf de Boer, Diana Delnoij

**Affiliations:** ^1^ Tranzo (Scientific Centre for Transformation in Care and Welfare)Tilburg UniversityTilburgThe Netherlands; ^2^ NIVEL (Netherlands Institute for Health Services Research)UtrechtThe Netherlands

**Keywords:** patient participation, patient‐reported outcome measures, questionnaire development procedures, scoping review

## Abstract

**Background:**

Patient‐reported outcome measures (PROMs) measure patients’ perspectives on health outcomes and are increasingly used in health care. To capture the patient's perspective, it is essential that patients are involved in PROM development

**Objective:**

This article reviews in what ways and to what extent patients are involved in PROM development and whether patient involvement has increased over time.

**Search strategy:**

Literature was searched in PubMed, EMBASE, MEDLINE and the Cochrane Methodology Register.

**Inclusion criteria:**

Studies were included if they described a new PROM development.

**Data extraction:**

Basic information and information regarding patient involvement in development phases was recorded.

**Main results:**

A total of 189 studies, describing the development of 193 PROMs, were included. Most PROMs were meant for chronic disease patients (*n* = 59) and measured quality of life (*n* = 28). In 25.9% of the PROM development studies, no patients were involved. Patients were mostly involved during item development (58.5%), closely followed by testing for comprehensibility (50.8%), while patient involvement in determining which outcome to measure was minimal (10.9%). Some patient involvement took place in the development of most PROMs, but in only 6.7% patients were involved in all aspects of the development. Patient involvement did not increase with time.

**Conclusions:**

Although patient involvement in PROM development is essential to develop valid patient‐centred PROMs, patients are not always involved. When patients are involved, their level of involvement varies considerably. These variations suggest that further attention to building and/or disseminating consensus on requirements for patient involvement in PROM development is necessary.

## Introduction

Patient‐reported outcome measures (PROMs) assess health outcomes from the patients’ perspective.^1^, ^2^ PROMs were originally developed for the use in clinical research as a way to measure treatment effectiveness. However, PROMs are now increasingly used in clinical practice to monitor and to improve the care for individual patients and in health policy and management, for example in the English National Health Service (NHS), to measure the performance of health‐care providers^2^, ^3^ or by the American Centres for Medicare and Medicaid Services (CMS) to award incentive payments for eligible professionals.^4^ Patients are more and more considered to possess important experiential knowledge on health and health care, a source of information that is relevant for improving quality of care.^2^, ^3^, ^4^, ^5^

To truly capture the patient's perspective, it is essential that patients are involved in PROM development,^5^, ^6^, ^7^, ^8^, ^9^ as only patients can determine which health outcomes are relevant for them^9^, ^10^, ^11^ and whether the questionnaire captures these outcomes in a comprehensible and understandable manner.^12^, ^13^, ^14^, ^15^ Besides, if a questionnaire fails to represent the patients’ perspective, it may result in patients failing to complete the questionnaire and a negative impact on the validity.^16^ Only a few studies investigated patient involvement in the development of PROMs, and only for a small number of PROMs, or for a specific disease.^7^, ^17^ Nevertheless, there are concerns that there are many PROMs in use where no patients were involved during the development process. Haywood investigated the quality and acceptability of PROMs used in chronic fatigue syndrome/myalgic encephalomyelitis and found no clear evidence of patient involvement in the development of PROMs.^17^ Other research using interviews with patients uncovered conceptual difficulties and questionnaire design problems among a few well‐known PROMs which might have been prevented by involving patients in the development.^7^ It appears that although patient involvement is increasingly accepted as an essential part of the development process, we are still using PROMs which were developed without patient involvement.

Although patient involvement appears to be neglected in PROM selection and at least in the development of earlier PROMs, there is some agreement in the literature on how patients should be involved. There are several phases in the development where the input of patients can be used to create a more patient‐centred PROM. To ensure that the health outcomes and domains measured with PROMs are relevant for patients, patients may partake in identifying core outcomes,^10^, ^11^ for example by participating in a reference group or focus groups.^18^ After establishing which outcomes should be measured, patients should be involved in the generation of items by either focus groups or interviews.^12^, ^15^, ^18^ While interviews can be used to obtain a number of personal feelings and opinions on a subject, which can be especially useful for eliciting the opinions of minority groups, focus groups may be useful for obtaining opinions that are likely to reflect the majority.^1^, ^19^ Choosing between these methods may also be dependent upon the patient population or practical considerations.^20^ Item development may have some overlap with the phase where core outcomes are identified, because although they have different purposes, both could take place using the same qualitative methods. After item development, it is important to determine that the questionnaire is comprehensible and that the content is valid for patients.^12^, ^13^, ^14^, ^15^ Structured cognitive interviews are an established way to ensure this^12^, ^13^ by enabling researchers to determine how items are interpreted by potential respondents and how a response is formed.^13^, ^15^

It appears that there are at least three phases in which patients can be involved in the development of PROMs, determining important health outcomes, item generation and checking for comprehensibility and content validity. However, as mentioned before, PROM development procedures are not standardized regarding patient involvement and the few studies who looked at patient involvement in the development of PROMs reported variations in whether and to what extent patients are involved in the development.^7^, ^17^ As patient involvement is such an important factor in the development of PROMs,^5^, ^9^, ^10^, ^11^, ^12^, ^13^, ^14^, ^15^, ^21^ it is of the utmost importance that all the parties involved are aware of the level of patient involvement of a PROM if it comes to selecting a PROM or interpreting PROM results. Additionally, awareness of the current situation regarding patient involvement is necessary so that all parties can act accordingly to ensure the development and use of valid patient‐centred PROMs. Therefore, by conducting a scoping review, this study aims to review the level of patient involvement in PROMs development. We expect to find more patient involvement in recently developed PROMs as compared to older PROMs, as the importance of the patients’ perspective is increasingly stressed.^2^, ^3^, ^4^, ^5^ In summary, our research questions are as follows:

1. In what ways and to what extent have patients been involved in the development of PROMs?
2. Has patient involvement in the development of PROMs increased with time?

## Methods

### Scoping review

This study is a scoping review. Scoping reviews are used to give a unique overview, in this case of patient involvement in the development of PROMs. For a scoping review, studies are not excluded based on type of study (as long as a description of a PROM development was included), type of participants, type of health care or development techniques. Furthermore, studies are not assessed on aspects of methodological quality. Characteristics of a scoping review can be a lack of a narrow review questions, the inclusion of studies which have employed a range of data collection and analysis techniques, the quality of the included studies is not assessed and the subject has not been subjected to a review before.^22^ Although we did not conduct a systematic review, the PRISMA statement^23^ was followed where possible.

### Search strategy

The search was conducted on 15 May 2014 by author BW. The databases used were PubMed, the Cochrane Methodology register, MEDLINE and EMBASE. The search terms were the mesh terms: (‘Outcome Assessment (Health Care)/methods ‘[Mesh] OR proms[tiab] OR ‘patient reported outcome measures ‘[tiab]) AND (questionnaires[mesh] OR questionnaire*[tiab]).^1^ The terms were determined after an initial search of the literature and advice from a librarian with expertise in health services research and systematic reviews. Studies describing translations or alterations of PROMs were checked for references to original PROM development studies. Initially, all languages were included and no time restriction was used.

### Literature selection

#### Inclusion criteria

Studies were included if they described the development of a new PROM. A PROM is a questionnaire which measures patients’ perspectives on health outcomes.^1^, ^2^ We considered the development of a PROM the process from establishing which outcomes to measure until the psychometric testing. This includes determining which outcome to measure, item development and testing the questionnaire on comprehensibility. Studies were included if they described (a part of) these phases.

#### Exclusion criteria

Studies which describe the development of a short version, alteration or translation of one already existing questionnaire do not usually go through all the development phases and were therefore excluded. Short versions and adaptations were considered closely to ensure that only studies were excluded which adapted an existing PROM very slightly or shortened a PROM without making any fundamental changes.

#### Post hoc exclusion criteria

Studies were excluded if they did not concern a published manuscript or if the manuscript was written in a language other than English, German, Dutch or French. As only two manuscripts were written in a different language, the impact was limited.

#### Study eligibility

**Figure 1:**
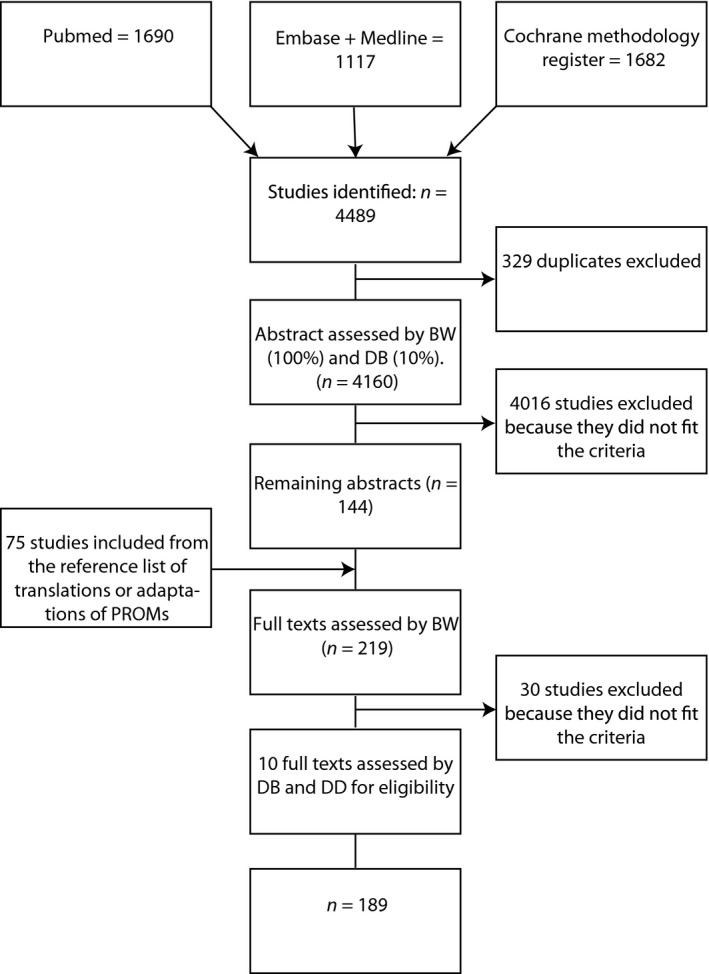
Search flow.

Author DB independently scored ten per cent of the abstracts on eligibility, after which disagreements were discussed. The inter‐rater agreement on study eligibility, calculated using the kappa statistic, was 0.71 (95% CI: [0.50, 0.92]). Full‐text selection by a second reviewer was deemed unnecessary as the reasons for exclusion were mostly very clear. However, authors DD and DB independently assessed the eligibility of 10 full texts. Additionally, full texts which were excluded because the questionnaire that was developed measured the process of care instead of outcomes, were discussed among the authors. There were no disagreements. See Fig. 1 for details on the literature selection process.

#### Data extraction

The basic information that was collected from each article included the first author, the year of publication, the country, the health problem or treatment associated with the PROM, whether the patient group concerned adults, parents or children, whether the PROM is generic or specific, the health outcome measured, and the care type associated with the health problem.

To gain insight into patient involvement in the development of PROMs, additional information was recorded which is displayed in Table 1.

**Table 1:** The categories for abstracting data regarding patient involvement

Category	Explanation	Example
**Determining which health outcome should be measured**	During this phase, it is determined which outcome is going to be measured, or domains or a framework are developed	
Patient involvement in determining which outcome is measured by including all the patients’ suggestions	Although patients were not actually asked which outcome should be measured, the patients’ suggestions were not restricted	‘Underlying the development of the questionnaire was a concept analysis and a description of patients’ postoperative recovery from the perspective of patients, registered nurses and surgeons’^42^
Patient involvement in determining which outcome is measured by letting patients help develop frameworks or domains	Patients were restricted to an outcome, but participated fully in developing domains or frameworks	‘As outlined in Fig. 1, the study involved four main steps: (1) qualitative concept elicitation interviews of children/adolescents with RLS and their parents…’^43^
**Item development**	During this phase, items are developed. This phase may have some overlap with the first phase as the same methods can be used for both framework development and item development. In case of overlap, both categories were scored	
The use of focus groups with patients	Focus groups with patients contributed to the development of items	‘Second step: to elicit relevant verbal material: Patients with hip or knee OA, and relevant health professionals, were recruited to take part in focus groups…’^44^
The use of interviews with patients	Interviews with patients contributed to the development of items	‘A pool of potential scale items was generated from semi‐structured interviews of 27 people with PSP’^45^
Patient involvement using other methods	Patients were involved in the item development using a different method than interviews or focus groups	‘Patients were first provided with an open‐ended free text space to comment broadly on what “quality of life” meant to them as they coped with MM’^46^
The use of other PROMs, literature or professionals	Items were developed (partly) using other sources than patients, such as experts, results of a literature review or other questionnaires	‘The Palliative Care Outcome Scale (POS) was developed using data from a review of other outcome measures used, or proposed for use in evaluating the palliative care of patients with advanced cancer’^47^
**Testing for comprehensibility**	During this phase, the developers ensure that the questionnaire is understandable and the questions are interpreted correctly	
The use of cognitive interviews with patients	Cognitive interviews with patients took place	‘Additionally, the questionnaire will be pretested with a variety of patients in cognitive interviews using the format outlined by Willis’^48^
The use of other methods involving patients	Patients were involved using a different method than cognitive interviews for testing the PROM for comprehensibility	‘The HDQoL was then pre‐tested on a group of 20 participants from pre‐symptomatic to late‐stage disease and reviewed in the light of their feedback’^49^
The use of professionals or other non‐patient groups	Other, non‐patient, groups were asked to assess the questionnaire for comprehensibility	‘To assess the questionnaire content validity (the extent to which a measurement reflects the specific intended domain of content and to test the items face validity (extent to which a measure seems to calculate a phenomenon on face value, or intuition, e.g. test/survey items are sent to experts to obtain suggestions for modification, comprehensiveness and relevance to haemophilia, a draft version of the questionnaire (75 items) was sent with a standardized evaluation form to a group of experts in the treatment of patients with haemophilia (*n* = 20) from the collaborating centres’^50^

Authors DD and DB extracted the data for 10 studies. Disagreements were discussed, after which the categories were further specified. Main discussion points were as follows:

1. Generic or specific? Initially, we defined a PROM as generic if everyone in the general population could answer the questions and a PROM as specific if it covered a specific disease. However, several PROMs were not generic, but covered health problems relevant for more than one disease, such as pain or fatigue. Conventional definitions^24^ would label this category as specific as it measures a specific concept. However, most of these questionnaires can be used for a far larger group of patients than a disease‐specific questionnaire could, which is why we gave this group of questionnaires the label ‘semi‐generic’.
2. What is regarded as patient involvement in determining which health outcome to measure? Although ideally patients are involved from the start of the development by asking them what outcomes are important to them, this is extremely rare. Therefore, we broadened this category to include cases where patients contributed to the development of the framework or domains within an established outcome or where patient suggestions were not restricted in any way.

### Statistics

A chi‐square test using the variables ‘Health outcome measured as outcome of surgery, cancer, chronic disease, mental health, other, lifelong disorder’ and ‘patient involvement (‘yes’ or ‘no’)’ was conducted to give insight into whether the level of patient involvement is influenced by the patient population for which the PROM is meant. To analyse patient involvement in PROM development over time, we distinguished between three time periods: before 2005 #bib2006–2010 and after 2010. The choice of these time frames was quite arbitrary, but it resulted in three groups of more or less equal size. The dependent variable described whether any patient involvement had taken place (‘yes’ or ‘no’). A chi‐square test was conducted to analyse whether patient involvement differs between these three time periods. *Post hoc* pair wise comparisons were conducted using a chi‐square test. Additional analyses were performed using different time periods: before and after 2006 (mean of PROM publication dates), before and after 2008 (median of PROM publication dates) and time as a continuous variable. Data were analysed using spss.^25^

## Results

### Search flow

As shown in Fig. 1 #bib4489 studies were identified from the databases PubMed, EMBASE, MEDLINE and the Cochrane methodology register. After a search for duplicates, 329 were removed, leaving 4160 studies. Of these studies, 219 studies remained after selecting abstracts which appeared to meet the inclusion criteria. Finally, a full‐text selection resulted in 189 studies. The studies excluded during full‐text selection were mostly presentations (*n* = 10), adaptations of already existing PROMs (*n* = 9) or patient‐reported experience measures (*n* = 4).

### Study characteristics

The 189 studies were published from 1980 until 2014 and described the development of 193 PROMs. The PROMs were mostly developed in the USA (*n* = 69), the UK (*n* = 62) and Germany (*n* = 17). Most PROMs were developed for a specific patient group (*n* = 152) or few patient groups (*n* = 32). Nine questionnaires were generic. Of the 193 PROMs, 180 questionnaires were meant to be filled out by adults and one by parents. A range of patient populations were included, from haemophilia (*n* = 6) and mental health patients (*n* = 5) to diabetes patients (*n* = 4). The majority of the PROMs were meant for chronic disease patients (*n* = 59) or for patients undergoing surgery (*n* = 25). Most PROMs measured solely quality of life (*n* = 28), health‐related quality of life (*n* = 21) or health status (*n* = 15). Both relatively unknown PROMs and much used PROMs such as the SF‐36, the EQ‐5D and the KOOS were included in the review.

### Patient involvement

In the development of 74.1% of PROM patients were involved in some way, leaving 25.9 per cent of PROMs without any patient involvement (Table 2). Patients were mostly involved in the item development (58.5%), especially using interviews (31.6%). Testing for comprehensibility closely followed item development (50.8%), while patients were never involved in determining which outcome should be measured. However, in some cases, patients were not limited to just the predetermined outcome and all suggestions were included (1.0%), and in some cases, patients were involved in developing the domains or a framework (9.8%). These examples of patient involvement together suggest some limited patient involvement in determining which outcome to measure in some PROMs (10.9%). In 6.7% of the PROM development studies, patients were involved in all the development phases we documented. The level of patient involvement did not differ dependent on whether the PROM was developed as a measurement of a health outcome of surgery, cancer, chronic disease, mental health or a lifelong disorder (χ^2^ (6) = 7.063, *P* = 0.315). There is a small significant difference between the levels of patient involvement for the three time periods (χ^2^ (2) = 6.511, *P* = 0.039). Patient involvement was significantly lower between the years 2006 and 2010 compared to after 2010 (χ^2^ (1) = 6.545, *P* = 0.011), but no significant difference was found between before 2005 and after 2010 (χ^2^ (1) = 2.872, *P* = 0.090) and between before 2005 and 2006–2010 (χ^2^ (1) = 1.048, *P* = 0.306). Sensitivity analyses using different time periods showed similar results.

**Table 2:** Patient involvement in the development of patient‐reported outcome measures (PROMs) (*n* = 193)

	*n*	%
**Patient involvement in any phase of the development** *	143	74.1
Patient involvement in one phase	67	34.7
Patient involvement in two phases	63	32.6
Patient involvement in three phases	13	6.7
**Establishing which outcome to measure**	21	10.9
Unrestricted input from patients	2	1.0
Patient involvement in the development of frameworks or domains	19	9.8
**Item development**	113	58.5
Focus groups with patients	42	21.8
Interviews with patients	61	31.6
Other methods involving patients	27	14.0
**Establishing the comprehensibility of the questionnaire**	98	50.8
Cognitive interviews	48	24.9
Other methods involving patients	55	28.5
Patient involvement before 2005	53	73.6
Patient involvement between 2006 and 2010	46	65.7
Patient involvement after 2010	44	86.3

*Questionnaire development was divided into three development phases: establishing which outcome to measure, item development and testing for comprehensibility.

## Discussion

As patient involvement in the development of PROMs is essential for measuring patient relevant outcomes,^5^, ^6^, ^7^, ^8^, ^9^ this study aimed to review whether and to what extent patients have been involved in the development of PROMs. In most PROM development studies, some patient involvement is recorded, but only in a few cases, patients have been involved throughout the development process. Surprisingly, in more than a quarter of PROM development studies, patient involvement was not documented at all. Other research looking at patient involvement in the development of PROMs for certain health problems suggests similar or even less favourable results for patient involvement.^7^, ^17^

Besides patient involvement in development phases, this review also investigated whether patient involvement increased with time. We expected that, as the patients’ perspective is increasingly regarded as important,^2^, ^3^, ^4^, ^5^ patients would be more involved in recently developed PROMs. There appeared to be some differences in patient involvement, but none that suggest an increase of patient involvement over time. This is especially surprising because patient involvement is not only recommended by many researchers,^5^, ^6^, ^7^, ^8^, ^9^ a few years ago it has also become a requirement if the PROM is used in medical product development.^26^ However, in ISOQOL's reporting standards for the use of PROMs in randomized controlled trials,^27^ the issue of patient involvement was rejected after consideration. Similarly, the COSMIN checklist, which assesses the methodological quality of studies on measurement properties of PROMs,^21^ barely mentions patient involvement. It only asks to check whether the items are relevant to the study population and whether the questionnaire was pre‐tested, for example using cognitive interviews. Apparently, patient involvement is still not always considered important enough to be included in reporting formats. Additionally, although many benefits may be gained by involving patients during the development process, patient involvement can also result in budget and time issues,^9^, ^28^ which may prevent PROM developers from involving patients. Differences in requirements regarding patient involvement and negative consequences of patient involvement may explain the variation we found in the level of patient involvement and the lack of a significant increase in patient involvement over time.

If patients are still not always involved, and if the involvement varies in intensity (from letting some patients comment on the questionnaire in writing after completing it, to the whole process of organizing focus groups, interviews and cognitive interviews), what does this mean for the validity of the questionnaires and for the use of their results by patients, health professionals and health insurers? Patient involvement is seen as essential to ensure the content validity of the questionnaire.^12^, ^13^, ^14^, ^15^ Involving patients by focus groups or interviews during item development ensures a better understanding of illness experience^20^ and could help challenge tacit models on which PROM designs are based.^29^ Furthermore, the content of the questionnaire may be less relevant to patients if they are not involved in the development. This, in turn, may lead to a negative attitude towards the questionnaire and failure to answer the questions. All this can negatively affect the validity of a survey.^16^ Based on the findings of this review, it appears that the content validity of many PROMs can be improved. This is troubling as PROMs are increasingly used to inform patients, to improve the care of individual patients and to assist purchasers.^2^, ^3^, ^30^, ^31^ PROMs may be used to improve the care of individual patients by increasing health professionals’ awareness of, and ability to, address patients’ concern.^32^ However, to use PROMs for this purpose, it is essential that a PROM is used which reflects the patients’ needs and concerns accurately.^33^ Furthermore, patient information based on PROMs may not sufficiently cover aspects that are most relevant to patients.

In the Netherlands, as well as in the English NHS and the American Medicare and Medicaid system, the use of PROMs in models of payment by results is a particularly relevant issue. In the Netherlands in 2006, managed competition has been introduced. In this system, health insurers should contract providers based on quality and costs of care. To do so, health insurers need instruments for measuring the quality of the care delivered by providers.^34^ Besides measures of effectiveness and safety and patient experience measures,^35^ PROMs are slowly introduced in the Netherlands to assist health insurers in selecting the best providers for contracting. In theory, this is a good thing, as PROMs are meant to reflect the patient's perspective^1^ and research suggests that patients have a different view on which aspects of their care are important to them. Identifying and understanding which aspects of care are the most important to patients may be the key to good care.^36^ However, if the validity of PROMs may need to be improved and if PROMs do not always reflect the patient's perspective, health insurers may want to ask themselves whether they are going to select health providers for the right reasons.

## Limitations

The present findings should be regarded with some caution because of study limitations. First, patient involvement was recorded using the documentation of the development process in the included articles. The results are therefore dependent on the quality and accuracy of the documentation. Patient involvement may go unnoticed if it is not documented in the article describing the development. Second, reporting standards may have improved over time and although patient involvement was recorded almost as many times in older publications as in recent publications, a lower standard of reporting may mean that levels of patient involvement in earlier studies have been underestimated. Third, not all development studies were documented in detail and sometimes PROM developers used one method for several development phases, which occasionally made it difficult to separate the development phases. However, to ensure that patient involvement was not underestimated, we recorded any suggestion that a method was used for more than one phase as patient involvement during several development phases. Fourth, as we wanted to compare PROMs on patient involvement during the three phases of development, we selected studies describing one or more of these phases. However, sometimes PROMs are further developed after the conclusion of these phases. As further development is very difficult to identify and the methods used for further development can vary greatly, we were unable to include this. Nevertheless, it is possible that for some of the PROMs included in this review, a higher level of patient involvement has been practised following the initial development. Fifth, although we were able to compare the PROMs on patient involvement in three development phases, we could not compare the PROMs on the level of patient involvement during these phases. The amount of information that is given in publications varies hugely and often lacks detailed information on patient involvement. Sixth, statistical analyses were performed on data which was not derived from a systematic review. Although systematic review guidelines were followed where possible, the included papers may not be a complete reflection of all PROM development papers, which may have implications for the interpretation of the results.

## Implications

This review may have several implications for the use and development of PROMs. First, a consensus should be reached on how patients should be involved in the development of PROMs. In this review several studies, describing opportunities to involve patients were brought together to use as a guide for extracting data concerning patient involvement from the papers. However, to ensure that all PROMs incorporate patient involvement throughout their development phases, it is important that one, easy to use, guideline is created. Perhaps inspiration may be gained from best practice examples such as the Genetic Counseling Outcome Scale^37^ and the Breast‐Q,^38^ where patients were involved throughout the development process. In the development of an instrument for fatigue in rheumatoid arthritis, patients were even given the role of research partners.^39^ Preferably the guideline is developed involving researchers, professionals, relevant organizations and patients to ensure input from all stakeholders and a widespread acceptation of the guideline.

Besides a guideline for patient involvement in PROM development, the implementation of a guideline for how to report the methods used to involve patients in publications is also necessary. As patient involvement is necessary to ensure that the questionnaire measures the patients’ perspective,^5^, ^6^, ^7^, ^8^, ^9^ existing PROMs should be selected based on the level of patient involvement. However, certain information about the development process is necessary for comparing PROMs on patient involvement. Although an attempt at guiding researchers in reporting patient involvement has been made,^40^ studies still offer varying amounts of information about their development process. This makes it very difficult to select a PROM based on its level of patient involvement. A guideline that is supported by all relevant parties and enforced by journals should help ensure that all publications include information needed to make an informed choice for a PROM.

Third, besides patient involvement in the development of PROMs, patients may also be involved in the interpretation and presentation of PROM results. Patients may be involved in the interpretation of PROM results using importance ratings. Importance ratings may give an indication of patient's values, needs and expectations regarding health care.^41^ Importance ratings enable the weighting of the PROM results according to the importance patients allocate towards aspects of the PROM. Furthermore, as patients can use PROM data to make an informed decision concerning health‐care providers or treatments,^30^, ^31^ PROM results should be freely available and easy to read. Patient involvement in the presentation of the results could help easy use of PROMs by patients.

## Conclusion

Although some patient involvement takes place in most PROM development studies, the level of patient involvement varies greatly. Furthermore, in more than a quarter of PROM development studies, no patient involvement was recorded. This lack of patient involvement throughout the development may have consequences for how well the questionnaire reflects the patient's perspective, which in turn may result in limited benefits for the use of PROMs in individual care, for making decisions by patients between treatments and health professionals and, finally, for rewarding health professionals or even selectively buying health care from certain health professionals. For PROMs to truly measure the patient's perspective, further attention to building and/or disseminating consensus on requirements for patient involvement in the development of PROMs is necessary and existing PROMs should be carefully selected on the level of patient involvement.

## Conflict of interest

The authors have no conflict of interests to disclose.

## Funding source

This study was funded by The National Health Care Institute, which is located in Diemen, the Netherlands. The National Health Care Institute did not have any role in the study design; in the collection, analysis and interpretation of data; in the writing of the report; and in the decision to submit the article for publication.

## Supporting information


**Appendix S1.** The results of the data abstraction from the included literature.

## Note

1 These search terms are related to the PubMed search. Similar versions were used to search the Cochrane Methodology register, MEDLINE and EMBASE but adapted for the search terms used in these databases.

## References

[1] Brédart A , Marrel A , Abetz‐Webb L , Lasch K , Acquadro C . Interviewing to develop Patient‐Reported Outcome (PRO) measures for clinical research: eliciting patients’ experience. Health and Quality of Life Outcomes, 2014; 12: 15. 24499454 10.1186/1477-7525-12-15 PMC3933509

[2] Black N . Patient reported outcome measures could help transform healthcare. BMJ, 2013; 346: f167. 23358487 10.1136/bmj.f167

[3] Devlin NJ , Parkin D , Browne J . Patient‐reported outcome measures in the NHS: new methods for analysing and reporting EQ‐5D data. Health Economics, 2010; 19: 886–905. 20623685 10.1002/hec.1608

[4] http://www.cms.gov/Regulations-andGuidance/Legislation/EHRIncentivePrograms/Downloads/EP_MeasuresTable_Posting_CQMs.pdf (accessed 31 December 2014).

[5] McKenna SP . Measuring patient‐reported outcomes: moving beyond misplaced common sense to hard science. BMC Medicine, 2011; 9: 86. 21756344 10.1186/1741-7015-9-86 PMC3170214

[6] Fitzpatrick R , Davey C , Buxton MJ , Jones DR . Evaluating patient‐based outcome measures for use in clinical trials. Health Technology Assessment, 1998; 2: 1–74. 9812244

[7] Paterson C . Seeking the patient's perspective: a qualitative assessment of EuroQol, COOP‐WONCA charts and MYMOP. Quality of Life Research, 2004; 13: 871–881. 15233501 10.1023/B:QURE.0000025586.51955.78

[8] Kirwan JR , Fries JF , Hewlett S , Osborne RH . Patient perspective: choosing or developing instruments. Journal of Rheumatology, 2011; 38: 1716–1719. 21807791 10.3899/jrheum.110390

[9] Staniszewska S , Adebajo A , Barber R *et al* Developing the evidence base of patient and public involvement in health and social care research: the case for measuring impact. International Journal of Consumer Studies, 2011; 35: 628–632.

[10] Haywood KL . Patient‐reported outcome II: selecting appropriate measures for musculoskeletal care. Musculoskeletal Care, 2007; 5: 72–90. 17542045 10.1002/msc.101

[11] Trujols J , Portella MJ , Iraurgi I , Campins MJ , Siñol N , de Los Cobos JP . Patient‐reported outcome measures: are they patient‐generated, patient‐centred or patient‐valued? Journal of Mental Health, 2013; 22: 555–562. 23323928 10.3109/09638237.2012.734653

[12] Coyne KS , Tubaro A , Brubaker L , Bavendam T . Development and validation of patient‐reported outcomes measures for overactive bladder: a review of concepts. Urology, 2006; 68(2 Suppl.): 9–16. 10.1016/j.urology.2006.05.042 16908336

[13] Patrick DL , Burke LB , Gwaltney CJ *et al* Content validity–establishing and reporting the evidence in newly developed patient‐reported outcomes (PRO) instruments for medical product evaluation: ISPOR PRO Good Research Practices Task Force report: part 2 – assessing respondent understanding. Value in Health, 2011; 14: 978–988. 22152166 10.1016/j.jval.2011.06.013

[14] Patrick DL , Burke LB , Gwaltney CJ *et al* Content validity–establishing and reporting the evidence in newly developed patient‐reported outcomes (PRO) instruments for medical product evaluation: ISPOR PRO good research practices task force report: part 1 – eliciting concepts for a new PRO instrument. Value in Health, 2011; 14: 967–977. 22152165 10.1016/j.jval.2011.06.014

[15] Turner R , Quittner AL , Parasuraman BM , Kallich JD , Cleeland CS , Mayo/FDA Patient‐Reported Outcomes Consensus Meeting Group . Patient‐reported outcomes: instrument development and selection issues. Value in Health, 2007; 10: S86–S93. 17995478 10.1111/j.1524-4733.2007.00271.x

[16] Meadows KA . Patient‐reported outcome measures: an overview. British Journal of Community Nursing, 2011; 16: 146–151. 21378658 10.12968/bjcn.2011.16.3.146

[17] Haywood KL , Staniszewska S , Chapman S . Quality and acceptability of patient‐reported outcome measures used in chronic fatigue syndrome/myalgic encephalomyelitis (CFS/ME): a systematic review. Quality of Life Research, 2012; 21: 35–52. 21590511 10.1007/s11136-011-9921-8

[18] Rose D , Evans J , Sweeney A , Wykes T . A model for developing outcome measures from the perspectives of mental health service users. International Review of Psychiatry, 2011; 23: 41–46. 21338297 10.3109/09540261.2010.545990

[19] Pesudovs K , Burr JM , Harley C , Elliott DB . The development, assessment, and selection of questionnaires. Optometry and Vision Science, 2007; 84: 663–674. 17700331 10.1097/OPX.0b013e318141fe75

[20] Streiner DL , Norman GR , Cairney J . Health Measurement Scales: A Practical Guide to Their Development and Use, 3rd edn New York: Oxford University Press, 2003.

[21] Mokkink LB , Terwee CB , Patrick DL *et al* The COSMIN checklist for assessing the methodological quality of studies on measurement properties of health status measurement instruments: an international Delphi study. Quality of Life Research, 2010; 19: 539–549. 20169472 10.1007/s11136-010-9606-8 PMC2852520

[22] Arksey H , O'Malley L . Scoping studies: towards a methodological framework. International Journal of Social Research Methodology, 2005; 8: 19–32.

[23] Liberati A , Altman DG , Tetzlaff J *et al* The PRISMA statement for reporting systematic reviews and meta‐analyses of studies that evaluate health care interventions: explanation and elaboration. Annals of Internal Medicine, 2009; 151: W‐65–W‐94. 10.7326/0003-4819-151-4-200908180-00136 19622512

[24] Patrick DL , Deyo RA . Generic and disease‐specific measures in assessing health status and quality of life. Medical Care, 1989; 27(3 Suppl): S217–S232. 2646490 10.1097/00005650-198903001-00018

[25] IBM Corp . Released 2010. IBM SPSS Statistics for Windows, Version 19.0. Armonk, NY: IBM Corp.

[26] U.S. Food and Drug Administration . Guidance for Industry Patient‐Related Outcome Measures: Use in Medical Product Development to Support Labeling Claims. Washington, DC: FDA, 2009.

[27] Brundage M , Blazeby J , Revicki D *et al* Patient‐reported outcomes in randomized clinical trials: development of ISOQOL reporting standards. Quality of Life Research, 2013; 22: 1161–1175. 22987144 10.1007/s11136-012-0252-1 PMC3731511

[28] Staniszewska S . Patient and public involvement in health services and health research: a brief overview of evidence, policy and activity. Journal of Research in Nursing, 2009; 14: 295–298.

[29] Staniszewska S , Haywood KL , Brett J , Tutton L . Patient and public involvement in patient‐reported outcome measures: evolution not revolution. The Patient, 2012; 5: 79–87. 22428752 10.2165/11597150-000000000-00000

[30] Burge P , Devlin N , Appleby J , Gallo F , Nason E , Ling T . Understanding Patients’ Choices at the Point of Referral.Technical report TR359‐DOH. Cambridge: RAND Europe, 2006.

[31] Wu AW , Snyder C , Clancy CM , Steinwachs DM . Adding the patient perspective to comparative effectiveness research. Health Affairs, 2010; 29: 1863–1871. 20921487 10.1377/hlthaff.2010.0660

[32] Marshall S , Haywood K , Fitzpatrick R . Impact of patient reported outcome measures on routine practice: a structured review. Journal of Evaluation in Clinical Practice, 2006; 12: 559–568. 16987118 10.1111/j.1365-2753.2006.00650.x

[33] Carr A , Higginson IJ . Measuring quality of life: are quality of life measures patient centred? BMJ, 2001; 322: 1357. 11387189 10.1136/bmj.322.7298.1357 PMC1120435

[34] Enthoven A , van de Ven WP . Going Dutch – managed‐competition health insurance in the Netherlands. The New England Journal of Medicine, 2007; 357: 2421–2423. 18077805 10.1056/NEJMp078199

[35] Delnoij D , Rademakers JJ , Groenewegen PP . The Dutch consumer quality index: an example of stakeholder involvement in indicator development. BMC Health Services Research, 2010; 10: 88. 20370925 10.1186/1472-6963-10-88 PMC2864255

[36] Land L , Hathorn E , Ross J . Using patient experience to measure the quality of HIV care. International Journal of STD & AIDS, 2011; 22: 366–367. 21729952 10.1258/ijsa.2011.010319

[37] McAllister M , Wood AM , Dunn G , Shiloh S , Todd C . The Genetic Counseling Outcome Scale: a new patient‐reported outcome measure for clinical genetics services. Clinical Genetics, 2011; 79: 413–424. 21255005 10.1111/j.1399-0004.2011.01636.x

[38] Pusic AL , Klassen AF , Scott AM , Klok JA , Cordeiro PG , Cano SJ . Development of a new patient‐reported outcome measure for breast surgery: the BREAST‐Q. Plastic and Reconstructive Surgery, 2009; 124: 345–353. 19644246 10.1097/PRS.0b013e3181aee807

[39] Nicklin J , Cramp F , Kirwan J , Urban M , Hewlett S . Collaboration with patients in the design of patient‐reported outcome measures: capturing the experience of fatigue in rheumatoid arthritis. Arthritis Care & Research, 2010; 62: 1552–1558. 20496429 10.1002/acr.20264

[40] Staniszewska S , Brett J , Mockford C , Barber R . The GRIPP checklist: strengthening the quality of patient and public involvement reporting in research. International Journal of Technology Assessment in Health Care, 2011; 27: 391–399. 22004782 10.1017/S0266462311000481

[41] Gourdji I , McVey L , Loiselle C . Patients’ satisfaction and importance ratings of quality in an outpatient oncology center. Journal of Nursing Care Quality, 2003; 18: 43–55. 12518838 10.1097/00001786-200301000-00007

[42] Allvin R , Ehnfors M , Rawal N , Svensson E , Idvall E . Development of a questionnaire to measure patient reported postoperative recovery: content validity and intra‐patient reliability. Journal of Evaluation in Clinical Practice, 2009; 15: 411–419. 19366398 10.1111/j.1365-2753.2008.01027.x

[43] Arbuckle R , Abetz L , Durmer JS *et al* Development of the Pediatric Restless Legs Syndrome Severity Scale (P‐RLS‐SS): a patient‐reported outcome measure of pediatric RLS symptoms and impact. Sleep Medicine, 2010; 11: 897–906. 20801715 10.1016/j.sleep.2010.03.016

[44] Rat AC , Coste J , Pouchot J *et al* OAKHQOL: a new instrument to measure quality of life in knee and hip osteoarthritis. Journal of Clinical Epidemiology, 2005; 58: 47–55. 15649670 10.1016/j.jclinepi.2004.04.011

[45] Schrag A , Selai C , Quinn N *et al* Measuring quality of life in PSP: the PSP‐QoL. Neurology, 2006; 67: 39–44. 16832075 10.1212/01.wnl.0000223826.84080.97

[46] Wagner LI , Robinson D Jr , Weiss M *et al* Content development for the Functional Assessment of Cancer Therapy‐Multiple Myeloma (FACT‐MM): use of qualitative and quantitative methods for scale construction. Journal of Pain and Symptom Management, 2012; 43: 1094–1104. 22575718 10.1016/j.jpainsymman.2011.06.019 PMC3419469

[47] Hearn J , Higginson IJ . Development and validation of a core outcome measure for palliative care: the palliative care outcome scale. Palliative Care Core Audit Project Advisory Group. Quality in Health Care, 1999; 8: 219–227. 10847883 10.1136/qshc.8.4.219 PMC2483665

[48] Morley D , Dummett S , Kelly L , Dawson J , Fitzpatrick R , Jenkinson C . The Oxford Participation and Activities Questionnaire: study protocol. Patient Related Outcome Measures, 2013; 5: 1–6. 24399888 10.2147/PROM.S53762 PMC3865144

[49] Hocaoglu MB , Gaffan EA , Ho AK . The Huntington's Disease health‐related Quality of Life questionnaire (HDQoL): a disease‐specific measure of health‐related quality of life. Clinical Genetics, 2012; 81: 117–122. 22151007 10.1111/j.1399-0004.2011.01823.x PMC3320660

[50] Arranz P , Remor E , Quintana M *et al* Development of a new disease‐specific quality‐of‐life questionnaire to adults living with haemophilia. Haemophilia, 2004; 10: 376–382. 15230953 10.1111/j.1365-2516.2004.00918.x

