# Supplementary material for: Patient involvement in the development of patient‐reported outcome measures: a scoping review
Source: Health Expect. 2016 Feb 18;20(1):11–23. doi: 10.1111/hex.12442 (PMC5217930; doi:10.1111/hex.12442)
Supplement: Supplementary file 1 — Appendix S1. The results of the data abstraction from the included literature. [file HEX-20-11-s001.docx]

| Author | Publication year | Country | Health problem | (Health) outcome | Patient group | Specific (1) or generic (2) or semi-generic (3) PROM | Health outcome measured as outcome of surgery (1), cancer (2), chronic disease (3), mental health(4), other (5), lifelong disorder (6) | Abbreviation | Patient involvement in establishing which outcome to measure (No=0; Yes =1; Limited = 2) | Patient involvement in item development | | | | Patient involvement in testing for comprehensibility | | | Patient involvement  (No=0; Yes =1) | The number of development phases with patient involvement |
| --- | --- | --- | --- | --- | --- | --- | --- | --- | --- | --- | --- | --- | --- | --- | --- | --- | --- | --- |
|  |  |  |  |  |  |  |  |  |  | Patient involvement, by use of focus groups, in item development  (No=0; Yes =1) | Patient involvement, by use of interviews, in item development  (No=0; Yes =1) | Patient involvement in item development using other methods  (No=0; Yes =1) | Items (partly) derived from other PROMs or other non-patient sources (No=0; Yes =1) | Cognitive interviews  (No=0; Yes =1) | Establishing the comprehensibility of the PROM by other means than cognitive interviews, but with the use of patients (No=0; Yes =1) | Establishing the comprehensibility of the PROM without patient input (No=0; Yes =1) |  |  |
| Meenan, RF ^1^ | 1980 | USA | Arthritis | Health status | Adults | 1 | 3 | AIMS | 0 | 0 | 0 | 0 | 1 | 0 | 0 | 0 | 0 | 0 |
| Hunt, SM ^2^ | 1980 | England | Generic | Health status | Adults | 2 | - | - | 1 | 0 | 1 | 0 | 0 | 1 | 1 | 1 | 1 | 3 |
| Neff, EJ ^3^ | 1990 | USA | Chronic diseases | Quality of life | Children | 3 | 3 | - | 0 | 0 | 1 | 1 | 0 | 0 | 0 | 0 | 1 | 1 |
| Corso, DM ^4^ | 1992 | USA | Medication use | Symptoms | Adults | 3 | 5 | - | 0 | 0 | 0 | 0 | 1 | 0 | 0 | 0 | 0 | 0 |
| Ware Jr, JE ^5^ | 1992 | USA | Generic | Health status | Adults (from 14 years) | 2 | - | SF-36 | 0 | 0 | 0 | 0 | 1 | 0 | 0 | 0 | 0 | 0 |
| Cella, DF ^6^ | 1993 | USA | Cancer | Quality of life | Adults | 1 | 2 | FACT | 0 | 0 | 1 | 0 | 0 | 0 | 0 | 0 | 1 | 1 |
| Garratt, AM ^7^ | 1993 | Scotland | Varicose veins | Pain and dysfunction, cosmetic appearance, extent of varicosity and complications. | Adults | 1 | 5 | AVVQ | 0 | 0 | 0 | 0 | 1 | 0 | 1 | 0 | 1 | 1 |
| Levine, DW ^8^ | 1993 | USA | Carpal tunnel syndrome | Severity of symptoms and functional status | Adults | 1 | 5 | - | 2 | 0 | 0 | 1 | 1 | 0 | 0 | 0 | 1 | 2 |
| Harwood, RH ^9^ | 1994 | England | Handicap | Handicap severity | Adults | 3 | 6 | - | 0 | 0 | 0 | 0 | 1 | 0 | 1 | 1 | 1 | 1 |
| Hays, RD ^10^ | 1994 | USA | Kidney disease | Quality of life | Adults | 1 | 3 | KDQOLTM | 0 | 1 | 0 | 0 | 1 | 0 | 0 | 0 | 1 | 1 |
| Ruta, DA ^11^ | 1994 | Scotland | Low back pain | Health status | Adults | 1 | 5 | - | 0 | 0 | 1 | 0 | 1 | 0 | 1 | 0 | 1 | 2 |
| Eisen, SV ^12^ | 1994 | USA | Mental health | Behaviour and symptoms | Adults | 3 | 4 | BASIS-32 | 0 | 0 | 0 | 1 | 0 | 0 | 0 | 0 | 1 | 1 |
| Barry, MJ ^13^ | 1995 | USA | Benign prostatic hyperplasia | Disease impact | Adults | 1 | 5 | BII | 0 | 0 | 0 | 0 | 1 | 0 | 1 | 0 | 1 | 1 |
| Barry, MJ ^13^ | 1995 | USA | Benign prostatic hyperplasia | Symptoms | Adults | 1 | 5 | SPI | 0 | 0 | 0 | 0 | 1 | 0 | 1 | 0 | 1 | 1 |
| Kolotkin, RL ^14^ | 1995 | USA | Obesity | Health-related quality of life | Adults | 1 | 5 | IWQOL | 0 | 1 | 1 | 0 | 0 | 0 | 1 | 0 | 1 | 2 |
| Kopec, JA ^15^ | 1995 | Canada | Back pain | Functional disability | Adults | 3 | 5 | - | 0 | 0 | 0 | 1 | 0 | 0 | 1 | 0 | 1 | 2 |
| Peto, V ^16^ | 1995 | England | Parkinson’s disease | Health status | Adults | 1 | 3 | PDQ-39 | 0 | 0 | 1 | 0 | 0 | 0 | 1 | 0 | 1 | 2 |
| Daltroy, LH ^17^ | 1996 | USA | Lumbar spine | Back pain related disability, neurogenic symptoms | Adults | 1 | 1 | - | 0 | 0 | 0 | 0 | 0 | 0 | 1 | 1 | 1 | 1 |
| Martin, DP ^18^ | 1996 | USA | Musculoskeletal disorders of the extremities | Health status | Adults | 1 | 5 | MFA | 0 | 0 | 1 | 0 | 1 | 0 | 0 | 0 | 1 | 1 |
| Hudak, PL ^19^ | 1996 | Canada | Upper extremity musculoskeletal conditions | Symptoms and functional status | Adults | 1 | 5 | DASH | 0 | 0 | 0 | 0 | 1 | 0 | 1 | 1 | 1 | 1 |
| Dawson, J ^20^ | 1996 | England | Total hip replacement | Problems related to the hip replacement | Adults | 1 | 1 | - | 0 | 0 | 1 | 0 | 1 | 0 | 1 | 0 | 1 | 2 |
| Dawson, J ^21^ | 1996 | England | Shoulder surgery related to a degenerative or inflammatory condition | Problems related to the shoulder surgery | Adults | 1 | 1 | - | 0 | 0 | 1 | 0 | 1 | 0 | 1 | 0 | 1 | 2 |
| Lambert, MJ ^22^ | 1996 | USA | Mental health | Subjective discomfort, interpersonal relations, and social role performance | Adults | 1 | 4 | OQ | 0 | 0 | 0 | 0 | 1 | 0 | 0 | 0 | 0 | 0 |
| Sprangers, MA ^23^ | 1996 | The Netherlands | Breast cancer | Quality of life | Adults | 1 | 2 | QLQ-BR23 | 0 | 0 | 1 | 0 | 1 | 0 | 1 | 0 | 1 | 2 |
| Stucki, G ^24^ | 1996 | Switzerland | Lumbar spinal stenosis | Symptom severity, physical function, and satisfaction | Adults | 1 | 1 | - | 0 | 0 | 0 | 0 | 1 | 0 | 1 | 0 | 1 | 1 |
| Fletcher, AE ^25^ | 1997 | England | Cataract | Vision function | Adults | 1 | 1 | - | 0 | 0 | 0 | 0 | 1 | 0 | 1 | 0 | 1 | 1 |
| Fletcher, AE ^25^ | 1997 | England | Cataract | Quality of life | Adults | 1 | 1 | - | 0 | 0 | 0 | 0 | 1 | 0 | 1 | 0 | 1 | 1 |
| The WHOQOL GROUP ^26^ | 1998 | Scotland | Generic | Quality of life | Adults | 2 | - | WHOQOL | 0 | 1 | 0 | 0 | 1 | 0 | 0 | 0 | 1 | 1 |
| Irrgang, JJ ^27^ | 1998 | USA | Knee disorders | Functional limitations imposed by pathological disorders and impairments of the knee during activities of daily living | Adults | 1 | 5 | - | 0 | 0 | 0 | 0 | 1 | 0 | 0 | 0 | 0 | 0 |
| Dawson, J ^28^ | 1998 | England | Total knee replacement | Knee replacement related problems | Adults | 1 | 1 | - | 0 | 0 | 1 | 0 | 0 | 0 | 1 | 0 | 1 | 2 |
| Deyo, RA ^29^ | 1998 | USA | Low back pain | Symptoms, function, general well-being, work disability and satisfaction with care | Adults | 1 | 5 | - | 0 | 0 | 0 | 0 | 1 | 0 | 0 | 0 | 0 | 0 |
| Roos, EM ^30^ | 1998 | Sweden | Knee injury and osteoarthritis | Pain, symptoms, activities of daily living, sport and recreation function, and knee-related quality of life | Adults | 1 | 5 | KOOS | 0 | 0 | 0 | 0 | 1 | 0 | 0 | 0 | 0 | 0 |
| Bradley, C ^31^ | 1999 | England | Diabetes | Quality of life | Adults | 1 | 3 | ADDQoL | 0 | 0 | 1 | 0 | 1 | 0 | 1 | 1 | 1 | 2 |
| Hawthorne, G ^32^ | 1999 | Australia | Generic | Health-related quality of life | Adults | 2 | - | AQoL | 0 | 0 | 0 | 0 | 1 | 1 | 0 | 1 | 1 | 1 |
| Haher, TR ^33^ | 1999 | USA | Idiopathic scoliosis | Quality of life | Adolescents | 1 | 1 | - | 0 | 0 | 0 | 0 | 1 | 0 | 0 | 0 | 0 | 0 |
| Hearn, J ^34^ | 1999 | England | Advanced cancer | Physical, psychological and spiritual domains of life within the remit of palliative care | Adults | 1 | 2 | POS | 0 | 0 | 0 | 1 | 1 | 0 | 1 | 0 | 1 | 2 |
| Varni, JW ^35^ | 1999 | USA | Chronic diseases | Health-related quality of life | Children | 3 | 3 | PedsQL | 0 | 0 | 1 | 0 | 1 | 0 | 1 | 0 | 1 | 2 |
| Younossi, ZM ^36^ | 1999 | USA | Chronic liver disease | Health-related quality of life | Adults | 1 | 3 | CLDQ | 0 | 1 | 1 | 0 | 1 | 1 | 0 | 0 | 1 | 2 |
| Garrow, AP ^37^ | 2000 | England | Foot-related problems | Foot pain and disability | Adults | 3 | 5 | - | 0 | 0 | 1 | 0 | 0 | 0 | 0 | 0 | 1 | 1 |
| Cox, R ^38^ | 2000 | USA | The use of hearing aids for hearing loss within the mild-to-severe range | ? | Adults | 1 | 5 | IOI-HA | 0 | 0 | 0 | 0 | 1 | 0 | 0 | 0 | 0 | 0 |
| Evans, C ^39^ | 2000 | England | Mental health | Well-being, problems/ symptoms, life functioning and risk to self and others | Adults | 1 | 4 | CORE-OM | 0 | 0 | 0 | 0 | 1 | 0 | 1 | 1 | 1 | 1 |
| Firth, H ^40^ | 2000 | England | Child psychiatric hospital admission | Worry and concern | Parents | 1 | 4 | - | 0 | 0 | 0 | 0 | 1 | 0 | 0 | 0 | 0 | 0 |
| Vitale, S ^41^ | 2000 | USA | Refractive Error | Vision-related quality of life | Adults | 1 | 5 | RSVP | 0 | 1 | 1 | 0 | 1 | 0 | 0 | 0 | 1 | 1 |
| Hobart, J ^42^ | 2001 | England | Multiple sclerosis | Physical and psychological impact | Adults | 1 | 3 | MSIS-29 | 0 | 0 | 1 | 0 | 1 | 0 | 0 | 0 | 1 | 1 |
| Morgan, MB ^43^ | 2001 | England | Chronic lower limb ischemia | Disease-specific quality of life | Adults | 1 | 3 | VascuQol | 0 | 1 | 0 | 0 | 1 | 0 | 1 | 0 | 1 | 2 |
| Rabin, R ^44^ | 2001 | The Netherlands | Generic | Health status | Adults | 2 | - | EQ-5D | 0 | 0 | 0 | 0 | 1 | 0 | 0 | 1 | 0 | 0 |
| Vickery, CW ^45^ | 2001 | England | Gastric cancer | Quality of life | Adults | 1 | 2 | QLQ-STO22 | 0 | 0 | 1 | 0 | 1 | 1 | 0 | 0 | 1 | 2 |
| Bialocerkowski, AE ^46, 47^ | 2002 | Australia | Wrist disorders | Activity limitation | Adults | 1 | 5 | - | 0 | 0 | 1 | 0 | 1 | 0 | 1 | 0 | 1 | 2 |
| Wilson, JTL ^48^ | 2002 | Scotland | Head injury | Disability | Adults | 1 | 5 | GOS | 0 | 0 | 0 | 0 | 1 | 0 | 0 | 0 | 0 | 0 |
| Spies, JB ^49^ | 2002 | USA | Leiomyomata | Health-related quality of life | Adults | 1 | 5 | UFS-QOL | 0 | 1 | 0 | 0 | 1 | 0 | 1 | 0 | 1 | 2 |
| Kazis, LE ^50^ | 2002 | USA | Burns | Health status | Children | 1 | 5 | - | 0 | 0 | 0 | 0 | 1 | 0 | 0 | 0 | 0 | 0 |
| Lamping, DL ^51^ | 2002 | England | Community-acquired pneumonia | Bothersomeness of symptoms | Adults | 1 | 5 | CAP-Sym | 2 | 0 | 1 | 0 | 0 | 0 | 0 | 1 | 1 | 2 |
| Manterola, C ^52^ | 2002 | Chile | Gastroesophageal reflux disease | Symptoms | Adults | 1 | 5 | - | 0 | 0 | 0 | 0 | 1 | 0 | 0 | 0 | 0 | 0 |
| Thompson, DR ^53^ | 2002 | England | Myocardial infarction | Health status | Adults | 1 | 5 | MIDAS | 0 | 0 | 1 | 0 | 0 | 0 | 0 | 0 | 1 | 1 |
| Kosinski, M ^54^ | 2003 | USA | Headache | Impact | Adults | 1 | 5 | HIT-6 | 0 | 0 | 0 | 0 | 1 | 0 | 0 | 0 | 0 | 0 |
| Macran, S ^55^ | 2003 | England | Podiatry | Health status | Adults | 3 | 5 | PHQ | 0 | 0 | 0 | 0 | 1 | 0 | 0 | 0 | 0 | 0 |
| Arranz, P ^56^ | 2004 | Spain | Haemophilia | Health-related quality of life | Adults | 1 | 6 | Haemophilia-QOL | 2 | 0 | 1 | 0 | 0 | 0 | 0 | 1 | 1 | 2 |
| Chasan-Taber, L ^57^ | 2004 | USA | Pregnancy | Physical activity | Adults | 1 | 5 | PPAQ | 0 | 0 | 1 | 0 | 0 | 0 | 0 | 0 | 1 | 1 |
| McMillan, CV ^58^ | 2004 | England | Hypothyroidism | Quality of life | Adults | 1 | 5 | ThyDQoL | 0 | 0 | 1 | 0 | 1 | 1 | 0 | 0 | 1 | 2 |
| McMillan, CV ^58^ | 2004 | England | Hypothyroidism | Treatment satisfaction | Adults | 1 | 5 | ThyTSQ | 0 | 0 | 1 | 0 | 1 | 1 | 0 | 0 | 1 | 2 |
| Haley, SM ^59^ | 2004 | USA | Post-acute care | Activity | Adults | 3 | 5 | AM-PAC | 0 | 1 | 0 | 0 | 1 | 0 | 0 | 0 | 1 | 1 |
| Mystakidou, K ^60^ | 2004 | Greece | Terminal cancer | Quality of life | Adults | 1 | 2 | PQLI | 0 | 0 | 0 | 1 | 1 | 0 | 1 | 1 | 1 | 2 |
| McKenna, SP ^61^ | 2004 | England | Psoriatic arthritis | Quality of life | Adults | 1 | 3 | PsAQoL | 0 | 0 | 1 | 0 | 0 | 1 | 0 | 0 | 1 | 2 |
| Young, NL ^62^ | 2004 | Canada | Haemophilia | Health-related quality of life | Children | 1 | 6 | CHO-KLAT | 0 | 1 | 0 | 1 | 1 | 1 | 0 | 1 | 1 | 2 |
| Spertus, J ^63^ | 2004 | USA | Peripheral arterial disease | Health status | Adults | 1 | 5 | PAQ | 0 | 0 | 1 | 0 | 1 | 0 | 0 | 0 | 1 | 1 |
| Van Genderen, FR ^64^ | 2004 | The Netherlands | Haemophilia | Health status | Adults | 1 | 6 | HAL | 0 | 0 | 0 | 1 | 0 | 0 | 1 | 0 | 1 | 2 |
| Becker, S ^65^ | 2005 | Germany | Dementia | Quality of life | Adults | 1 | 3 | H.I. L.DE. | 0 | 0 | 1 | 0 | 1 | 0 | 0 | 0 | 1 | 1 |
| Chen, CC ^66^ | 2005 | USA | Hand impairments | Manual ability | Adults | 3 | 5 | MAM-16 | 0 | 0 | 0 | 0 | 1 | 0 | 0 | 1 | 0 | 0 |
| Malaty, HM ^67^ | 2005 | USA | Recurrent abdominal pain | Pain intensity, non-pain symptoms, pain disability, and satisfaction with health | Children | 1 | 5 | MM-RAP | 0 | 0 | 0 | 0 | 1 | 0 | 0 | 0 | 0 | 0 |
| Rat, AC ^68^ | 2005 | France | Knee and hip osteoarthritis | Quality of life | Adults | 1 | 3 | OAKHQOL | 2 | 1 | 1 | 1 | 1 | 0 | 0 | 0 | 1 | 2 |
| Smith, SC ^69^ | 2005 | England | Dementia | Health-related quality of life | Adults | 1 | 3 | DEMQOL | 2 | 0 | 0 | 0 | 1 | 1 | 0 | 0 | 1 | 2 |
| Vidhubala, E ^70^ | 2005 | India | Cancer | Quality of life | Adults | 1 | 2 | - | 0 | 0 | 0 | 0 | 1 | 0 | 0 | 1 | 0 | 0 |
| Schaefer, AM ^71^ | 2006 | England | Mitochondrial diseases | Current function, system specific involvement, current clinical assessment; and quality of life | Adults | 1 | 5 | NMDAS | 0 | 0 | 0 | 0 | 1 | 0 | 0 | 0 | 0 | 0 |
| Atkinson, MJ ^72^ | 2006 | USA | Oily skin on the face and scalp | Symptomatic impact | Adults | 1 | 5 | - | 0 | 1 | 0 | 0 | 0 | 1 | 0 | 0 | 1 | 2 |
| Cano, SJ ^73^ | 2006 | England | Plastic surgery for head/neck skin lesions in patients with skin cancer | Symptoms, psychological functioning, limitations of daily activities, cosmetic appearance and patient satisfaction | Adults | 1 | 1 | POS-Head/Neck | 2 | 0 | 1 | 0 | 0 | 0 | 1 | 0 | 1 | 3 |
| Chen, TYT ^74^ | 2006 | New Zealand | Gallstone disease | Quality of life | Adults | 1 | 1 | CSQ | 0 | 0 | 0 | 0 | 1 | 0 | 1 | 0 | 1 | 1 |
| Urbach, DR ^75^ | 2006 | Canada | Abdominal surgery | Health-related quality of life | Adults | 3 | 1 | - | 2 | 0 | 0 | 0 | 1 | 0 | 0 | 0 | 1 | 1 |
| Colwell, HH ^76^ | 2006 | USA | Gout | Pain, gout flares, health distress, days lost from work and treatment satisfaction | Adults | 1 | 3 | GAQ | 0 | 0 | 0 | 0 | 1 | 1 | 0 | 0 | 1 | 1 |
| McKenna, SP ^77^ | 2006 | England | Pulmonary hypertension | Health-related quality of life and quality of life | Adults | 1 | 3 | CAMPHOR | 0 | 0 | 1 | 0 | 1 | 1 | 0 | 0 | 1 | 2 |
| McMillan, CV ^78^ | 2006 | England | Hypopituitarism | Quality of life | Adults | 1 | 5 | HDQoL | 0 | 0 | 0 | 0 | 1 | 0 | 0 | 0 | 0 | 0 |
| Taback, NA ^79^ | 2006 | Canada | Genital herpes | Treatment satisfaction | Adults | 1 | 5 | GHerpTSQ | 0 | 0 | 0 | 0 | 1 | 0 | 0 | 0 | 0 | 0 |
| Nordyke, RJ ^80^ | 2006 | USA | Anaemia treatment | Patient satisfaction | Adults | 1 | 5 | PSQ-An | 0 | 0 | 0 | 0 | 1 | 0 | 0 | 0 | 0 | 0 |
| Osborne, RH ^81^ | 2006 | Australia | Chronic diseases | Health status | Adults | 3 | 3 | HEI-Q Perspective | 0 | 0 | 0 | 0 | 1 | 1 | 0 | 0 | 1 | 1 |
| Pollak, E ^82^ | 2006 | Germany | Haemophilia | Health-related quality of life | Children and adolescents | 1 | 6 | Haemo-QoL Index | 0 | 0 | 0 | 0 | 1 | 0 | 0 | 0 | 0 | 0 |
| Schrag, A ^83^ | 2006 | England | Progressive supranuclear palsy | Quality of life | Adults | 1 | 3 | PSP-QoL | 0 | 0 | 1 | 0 | 1 | 0 | 0 | 0 | 1 | 1 |
| Vasiliadis, E ^84^ | 2006 | Greece | Brace treated scoliosis | Quality of life | children | 1 | 5 | BRQ | 0 | 0 | 1 | 0 | 1 | 0 | 0 | 0 | 1 | 1 |
| Yaruss, JS ^85^ | 2006 | USA | Stuttering disorder | The speaker’s perceptions about stuttering, the speaker’s difficulties communicating in daily situations and the overall impact of stuttering on the speaker’s quality of life | Adults | 1 | 5 | OASES | 0 | 1 | 0 | 0 | 1 | 0 | 1 | 1 | 1 | 2 |
| Fukui, M ^86^ | 2007 | Japan | Cervical myelopathy | Health-related quality of life and physical functions | Adults | 1 | 5 | JOACMEQ | 0 | 0 | 0 | 0 | 1 | 0 | 0 | 0 | 0 | 0 |
| Gandek, B ^87^ | 2007 | USA | Post-acute care | Participation | Adults | 3 | 5 | PM-PAC | 0 | 0 | 0 | 0 | 0 | 1 | 1 | 0 | 1 | 1 |
| Nilsson-Helander, K ^88^ | 2007 | Sweden | Total Achilles tendon rupture | Symptoms and physical activity | Adults | 1 | 5 | ATRS | 0 | 0 | 0 | 0 | 1 | 0 | 1 | 0 | 1 | 1 |
| Osborne, RH ^89^ | 2007 | Australia | Chronic diseases | Positive and active engagement in life, health directed behaviour, skill and technique acquisition, constructive attitudes and approaches, self-monitoring and insight, health service navigation, social integration and support and emotional well-being | Adults | 3 | 3 | HeiQ | 0 | 0 | 0 | 0 | 1 | 0 | 0 | 0 | 0 | 0 |
| Rodrigues, G ^90^ | 2007 | Canada | Prostate cancer radiation late toxicity | Health-related quality of life | Adults | 1 | 2 | PCRT | 0 | 0 | 0 | 0 | 1 | 0 | 1 | 1 | 1 | 1 |
| Schrag, A ^91^ | 2007 | England | Multiple system atrophy | Health-related quality of life | Adults | 1 | 3 | MSA-QoL | 0 | 0 | 1 | 0 | 1 | 0 | 0 | 0 | 1 | 1 |
| Shirado, O ^92^ | 2007 | Japan | Chronic low back pain | Health-related quality of life, psychosomatic factors | Adults | 1 | 3 | JLEQ | 0 | 0 | 0 | 0 | 1 | 0 | 1 | 0 | 1 | 1 |
| Walter, OB ^93^ | 2007 | Germany | Anxiety | Anxiety | Adults | 3 | 4 | Anxiety-CAT | 0 | 0 | 0 | 0 | 1 | 0 | 0 | 0 | 0 | 0 |
| Arbuckle, R ^94^ | 2008 | England | Oily facial skin | Symptom severity | Adults | 1 | 5 | OSSAS | 2 | 1 | 0 | 0 | 1 | 1 | 0 | 0 | 1 | 3 |
| Arbuckle, R ^94^ | 2008 | England | Oily facial skin | Emotional impact | Adults | 1 | 5 | OSIS | 2 | 1 | 0 | 0 | 1 | 1 | 0 | 0 | 1 | 3 |
| Bouffioulx, E ^95^ | 2008 | Belgium | Chronic stroke | Satisfaction with activity and participation | Adults | 1 | 3 | SATIS-Stroke scale | 0 | 0 | 0 | 1 | 1 | 0 | 0 | 0 | 1 | 1 |
| Caty, GD ^96^ | 2008 | Belgium | Stroke | Locomotion ability | Adults | 1 | 5 | ABILOCO | 0 | 0 | 0 | 0 | 1 | 0 | 0 | 0 | 0 | 0 |
| Caty, GD ^97^ | 2008 | Belgium | Cerebral palsy | Locomotion ability | Children | 1 | 6 | ABILOCO-KIDS | 0 | 0 | 0 | 0 | 1 | 0 | 0 | 0 | 0 | 0 |
| Dawson, JH ^98^ | 2008 | England | Elbow surgery | Health status | Adults | 3 | 1 | - | 0 | 0 | 1 | 0 | 0 | 0 | 0 | 0 | 1 | 1 |
| Deering, DE ^99^ | 2008 | New Zealand | Methadone maintenance treatment | Recent substance use, aspects of social and behavioural functioning, and physical and psychological health | Adults | 1 | 5 | MTI | 0 | 0 | 0 | 0 | 1 | 0 | 0 | 0 | 0 | 0 |
| Kushner, JA ^100^ | 2008 | Canada | Oral Mucositis | Symptoms | Adults | 1 | 5 | - | 0 | 0 | 1 | 0 | 0 | 0 | 0 | 0 | 1 | 1 |
| McMillan, C ^101^ | 2008 | England | Hypothyroidism | Bothersomeness of symptoms | Adults | 1 | 5 | ThySRQ | 0 | 0 | 1 | 0 | 1 | 1 | 0 | 0 | 1 | 2 |
| Rentz, A ^102^ | 2008 | USA | Haemophilia | Health-related quality of life | Adults | 1 | 6 | HAEMO-QoL-A | 2 | 1 | 0 | 0 | 1 | 1 | 0 | 0 | 1 | 3 |
| Rose, M ^103^ | 2008 | USA | Generic | Physical function | Adults | 2 | - | - | 0 | 0 | 0 | 0 | 1 | 0 | 0 | 0 | 0 | 0 |
| Rosen, R ^104^ | 2008 | USA | Peyronie’s disease | Sexual and psychosocial functioning | Adults | 1 | 5 | - | 0 | 1 | 0 | 0 | 0 | 0 | 0 | 0 | 1 | 1 |
| Webster, KE ^105^ | 2008 | Australia | Anterior cruciate ligament reconstruction surgery | Psychological impact | Adults | 3 | 1 | - | 0 | 0 | 0 | 0 | 1 | 0 | 1 | 0 | 1 | 1 |
| Allvin, R ^106^ | 2009 | Sweden | Surgery | Postoperative recovery | Adults | 3 | 1 | - | 1 | 0 | 0 | 0 | 1 | 0 | 1 | 0 | 1 | 2 |
| Baumann, I ^107^ | 2009 | Germany | Parotidectomy for benign disease | Health-related quality of life | Adults | 1 | 1 | POI-8 | 0 | 0 | 0 | 0 | 1 | 0 | 0 | 0 | 0 | 0 |
| Baumann, I ^108^ | 2009 | Germany | Chronic otitis media | Health-related quality of life | Adults | 1 | 3 | COMOT-15 | 0 | 0 | 0 | 0 | 1 | 0 | 0 | 0 | 0 | 0 |
| Blome, C ^109^ | 2009 | Germany | Pruritus | Patient-relevant benefit | Adults | 1 | 5 | PBI-P | 0 | 0 | 0 | 1 | 1 | 0 | 0 | 0 | 1 | 1 |
| Blome, C ^110^ | 2009 | Germany | Chronic hand eczema | Patient-relevant benefit | Adults | 1 | 3 | PBI-HE | 0 | 0 | 0 | 1 | 1 | 0 | 0 | 0 | 1 | 1 |
| Brod, M ^111^ | 2009 | USA | Diabetes | Treatment impact | Adults | 1 | 3 | TRIM-Diabetes | 0 | 1 | 1 | 0 | 1 | 1 | 0 | 0 | 1 | 2 |
| Brod, M ^111^ | 2009 | USA | Diabetes | Treatment impact | Adults | 1 | 3 | TRIM-Diabetes Device | 0 | 1 | 1 | 0 | 1 | 1 | 0 | 0 | 1 | 2 |
| Doward, LC ^112^ | 2009 | England | Multiple sclerosis | Symptoms, activities, and quality of life | Adults | 1 | 3 | PRIMUS | 0 | 0 | 1 | 0 | 1 | 1 | 0 | 0 | 1 | 2 |
| Helbostad, JL ^113^ | 2009 | Norway | Palliative Cancer care | Physical functioning | Adults | 1 | 2 | - | 0 | 0 | 0 | 0 | 1 | 0 | 0 | 0 | 0 | 0 |
| Jette, AM ^114^ | 2009 | USA | Lower extremity osteoarthritis | Disability | Adults | 1 | 3 | OA-DISABILITY-CAT | 0 | 1 | 0 | 0 | 1 | 1 | 0 | 0 | 1 | 2 |
| Katz, JN ^115^ | 2009 | USA | Musculoskeletal problems | Functional limitations | Adults | 3 | 5 | - | 0 | 0 | 0 | 0 | 1 | 0 | 0 | 0 | 0 | 0 |
| Li, T ^116^ | 2009 | USA | Rheumatoid arthritis | Activity participation | Adults | 1 | 3 | APaQ | 0 | 0 | 0 | 0 | 1 | 1 | 0 | 0 | 1 | 1 |
| Pollard, B ^117^ | 2009 | Scotland | Osteoarthritis | Impairment | Adults | 1 | 3 | Ab-I | 0 | 0 | 0 | 0 | 1 | 0 | 0 | 0 | 0 | 0 |
| Pollard, B ^117^ | 2009 | Scotland | Osteoarthritis | Activity limitation | Adults | 1 | 3 | Ab-A | 0 | 0 | 0 | 0 | 1 | 0 | 0 | 0 | 0 | 0 |
| Pollard, B ^117^ | 2009 | Scotland | Osteoarthritis | Participation restriction | Adults | 1 | 3 | Ab-P | 0 | 0 | 0 | 0 | 1 | 0 | 0 | 0 | 0 | 0 |
| Pusic, AL ^118, 119^ | 2009 | USA | Breast surgery | Patient satisfaction and health-related quality of life | Adults | 3 | 1 | BREAST-Q | 2 | 1 | 1 | 0 | 1 | 1 | 0 | 0 | 1 | 3 |
| Shaikh, N ^120^ | 2009 | USA | Acute Otitis Media | Symptom burden | Children | 1 | 5 | AOM-SOS | 0 | 0 | 0 | 1 | 1 | 0 | 1 | 0 | 1 | 2 |
| Agarwal, R ^121^ | 2010 | USA | Chronic kidney disease | Presence and severity of symptoms | Adults | 1 | 3 | - | 0 | 0 | 1 | 0 | 1 | 0 | 0 | 0 | 1 | 1 |
| Aghayev, E ^122^ | 2010 | Switzerland | Generic | Sleep quality | Adults | 2 | - | SEQ-Sleep | 0 | 0 | 0 | 1 | 1 | 0 | 0 | 0 | 1 | 1 |
| Arbuckle, R ^123^ | 2010 | England | Paediatric restless legs syndrome | Symptoms and impact | Children | 1 | 6 | P-RLS-SS | 2 | 0 | 1 | 0 | 1 | 1 | 0 | 0 | 1 | 3 |
| Bishop, FL ^124^ | 2010 | England | Spiritual healing | Outlook, energy, health, relationships, emotional  balance | Adults | 3 | 5 | HEHIQ | 0 | 1 | 0 | 0 | 0 | 1 | 0 | 0 | 1 | 2 |
| Bode, RK ^125^ | 2010 | USA | Generic | Ability to participate and satisfaction with participation in social activities | Adults | 2 | - | - | 0 | 1 | 0 | 0 | 1 | 0 | 0 | 0 | 1 | 1 |
| Deal, LS ^126^ | 2010 | USA | Endometriosis | Symptoms | Adults | 1 | 3 | EPBD | 0 | 1 | 0 | 0 | 1 | 1 | 0 | 0 | 1 | 2 |
| Deal, LS ^127^ | 2010 | USA | Endometriosis | Treatment satisfaction | Adults | 1 | 3 | ETSQ | 0 | 1 | 0 | 0 | 1 | 1 | 0 | 0 | 1 | 2 |
| El Miedany, Y ^128^ | 2010 | England | Inflammatory arthritis | Functional disability and quality of life | Adults | 1 | 3 | - | 0 | 0 | 0 | 0 | 1 | 0 | 0 | 0 | 0 | 0 |
| El Miedany, Y ^129^ | 2010 | England | Ankylosing spondylitis/ spondyloarthritis | Functional disability, quality of life, VAS for spinal pain, joint pain, global status, fatigue, duration of morning stiffness, review of the systems, falls and cardiovascular risks, self-helplessness as well as self-reported joint and soft tissue pain | Adults | 1 | 3 | - | 0 | 0 | 0 | 0 | 1 | 0 | 0 | 0 | 0 | 0 |
| Haywood, KL ^130^ | 2010 | England | Ankylosing Spondylitis | Quality of life | Adults | 1 | 3 | EASi-QoL | 0 | 0 | 1 | 0 | 1 | 0 | 0 | 0 | 1 | 1 |
| Herlyn, K ^131^ | 2010 | Germany | Vasculitis | Burden of disease | Adults | 3 | 5 | - | 0 | 0 | 0 | 0 | 1 | 0 | 0 | 0 | 0 | 0 |
| Klassen, AF ^132^ | 2010 | Canada | Facial aesthetic problems | Satisfaction with facial appearance; health-related quality of life; recovery, early life impact, and adverse effects; and satisfaction with process of care | Adults | 3 | 5 | FACE-Q | 2 | 0 | 1 | 0 | 1 | 0 | 0 | 0 | 1 | 2 |
| Leidy, NK ^133^ | 2010 | USA | Chronic obstructive pulmonary disease | Frequency, severity, and duration of exacerbations | Adults | 1 | 3 | EXACT | 0 | 1 | 1 | 0 | 1 | 1 | 0 | 0 | 1 | 2 |
| Nicklin, J ^134^ | 2010 | England | Rheumatoid Arthritis | Fatigue | Adults | 1 | 3 | - | 2 | 1 | 0 | 0 | 1 | 1 | 0 | 0 | 1 | 3 |
| Von Mackensen, S ^135^ | 2010 | Germany | Haemophilia | Subjective physical performance | Adults | 1 | 6 | HEP-Test-Q | 0 | 0 | 0 | 0 | 1 | 0 | 1 | 0 | 1 | 1 |
| Von Steinbuchel, N ^136^ | 2010 | Germany | Traumatic brain injury | Health-related quality of life | Adults | 1 | 5 | QOLIBRI | 0 | 0 | 0 | 0 | 1 | 0 | 0 | 0 | 0 | 0 |
| Zimmerman, M ^137^ | 2010 | USA | Mental health | Anxiety | Adults | 3 | 4 | CUXOS | 0 | 0 | 0 | 0 | 1 | 0 | 0 | 0 | 0 | 0 |
| Gibbons, CJ ^138^ | 2011 | England | Motor neurone disease | Fatigue | Adults | 1 | 3 | NFI-MND | 0 | 0 | 1 | 0 | 1 | 0 | 1 | 0 | 1 | 2 |
| Jackson, MJ ^139^ | 2011 | England | Urethral Stricture Surgery | Symptoms and health-related quality of life | Adults | 1 | 1 | - | 0 | 0 | 1 | 0 | 1 | 0 | 0 | 1 | 1 | 1 |
| Matza, LS ^140^ | 2011 | USA | Depression | Fatigue | Adults | 1 | 4 | FAsD | 0 | 1 | 0 | 0 | 1 | 1 | 0 | 0 | 1 | 2 |
| McAllister, M ^141, 142^ | 2011 | England | Clinical genetics services | Empowerment | Adults | 1 | 5 | GCOS-24 | 2 | 1 | 1 | 0 | 1 | 1 | 1 | 0 | 1 | 3 |
| Ritenbaugh, C ^143^; Thompson, JJ ^144^ | 2011 | USA | Complementary and alternative medicine | Well-being | Adults | 3 | 5 | SAC | 0 | 0 | 1 | 1 | 1 | 1 | 0 | 0 | 1 | 2 |
| Amtmann, D ^145^ | 2012 | USA | Multiple sclerosis and spinal cord injury | Self-efficacy | Adults | 3 | 5 | UWSES | 0 | 0 | 0 | 0 | 1 | 0 | 0 | 0 | 0 | 0 |
| Drapalski, AL ^146^ | 2012 | USA | Serious mental illness | Recovery | Adults | 1 | 4 | MARS | 0 | 0 | 0 | 1 | 1 | 0 | 1 | 1 | 1 | 2 |
| Aufwerber, S ^147^ | 2012 | Sweden | Anterior cruciate ligament reconstruction | Donor-site-related functional problem | Adults | 1 | 1 | - | 0 | 0 | 0 | 0 | 1 | 0 | 1 | 0 | 1 | 1 |
| Chen, RQ ^148^ | 2012 | China | Traditional Chinese medicine syndromes kidney-yin deficiency syndrome and kidney-yang deficiency syndrome | Symptoms | Adults | 1 | 5 | KDSQ | 0 | 0 | 0 | 0 | 1 | 0 | 0 | 1 | 0 | 0 |
| Gabel, CP ^149^ | 2012 | Australia | Lower-extremity conditions | Function | Adults | 3 | 5 | LLFI | 0 | 1 | 0 | 0 | 1 | 0 | 0 | 0 | 1 | 1 |
| Govender, R ^150^ | 2012 | England | Total laryngectomy | Swallowing function | Adults | 3 | 1 | SOAL questionnaire | 0 | 1 | 0 | 0 | 1 | 0 | 1 | 0 | 1 | 2 |
| Hocaoglu, MB ^151^ | 2012 | England | Huntington’s disease | Health-related quality of life | Adults | 1 | 3 | HDQoL | 0 | 0 | 1 | 1 | 0 | 0 | 1 | 0 | 1 | 2 |
| Jolly, M ^152^ | 2012 | USA | Systemic lupus erythematosus | health and non-health-related quality of life | Adults | 1 | 3 | LupusPRO | 0 | 0 | 1 | 0 | 1 | 0 | 1 | 0 | 1 | 2 |
| Kaufmann, H ^153^ | 2012 | USA | Neurogenic orthostatic Hypotension | Symptom burden and severity | Adults | 1 | 5 | OHQ | 0 | 1 | 0 | 0 | 1 | 0 | 0 | 0 | 1 | 1 |
| Lasch, KE ^154^ | 2012 | USA | Major depressive disorder | Symptoms | Adults | 1 | 4 | - | 2 | 1 | 0 | 0 | 0 | 1 | 0 | 0 | 1 | 3 |
| Mohtadi, NGH ^155^ | 2012 | Canada | Symptomatic hip disease | Health-related quality of life | Adults | 3 | 5 | iHOT-33 | 0 | 0 | 1 | 1 | 1 | 0 | 1 | 1 | 1 | 2 |
| Mojtabai, R ^156^ | 2012 | USA | Schizophrenia and schizoaffective disorder | Report measure of psychiatric symptoms, medication side effects and general well-being | Adults | 1 | 4 | PAQ | 0 | 1 | 0 | 0 | 1 | 0 | 1 | 0 | 1 | 2 |
| Otter, SJ ^157^ | 2012 | England | Rheumatoid arthritis | Quality of life | Adults | 1 | 3 | - | 0 | 0 | 0 | 1 | 1 | 0 | 1 | 0 | 1 | 2 |
| Pinder, B ^158^ | 2012 | England | Intermittent self-catheterization for people with chronic urinary retention | Quality of life | Adults | 1 | 3 | ISC-Q | 0 | 0 | 1 | 0 | 1 | 1 | 0 | 0 | 1 | 2 |
| Schmidutz, F ^159^ | 2012 | Germany | Shoulder surgery | Patient safety and treatment efficiency | Adults | 3 | 1 | MSQ | 0 | 0 | 0 | 0 | 1 | 0 | 0 | 0 | 0 | 0 |
| Skevas, T ^160^ | 2012 | Germany | Tonsillectomy | Health-related quality of life | Adults | 1 | 1 | TOI-14 | 0 | 0 | 0 | 0 | 1 | 0 | 0 | 0 | 0 | 0 |
| Wagner, LI ^161^ | 2012 | USA | Multiple myeloma | Health-related quality of life | Adults | 1 | 2 | FACT-MM | 0 | 0 | 0 | 1 | 1 | 0 | 0 | 1 | 1 | 1 |
| Glaser, AW ^162^ | 2013 | England | Cancer survivors | Quality of life | Adults | 1 | 2 | - | 0 | 0 | 0 | 1 | 1 | 1 | 0 | 0 | 1 | 2 |
| Hareendran, A ^163^ | 2013 | USA | Chronic obstructive pulmonary disease | Night-time symptoms | Adults | 1 | 3 | NiSCI | 0 | 1 | 0 | 0 | 1 | 1 | 0 | 0 | 1 | 2 |
| Bankstahl, US ^164^ | 2013 | Germany | Subjective tinnitus | Cognitive impairment | Adults | 1 | 3 | APSA | 0 | 0 | 0 | 0 | 1 | 1 | 1 | 0 | 1 | 1 |
| Gorecki, C ^165^ | 2013 | England | Pressure ulcers | Health-related quality of life | Adults | 1 | 5 | PU-QOL | 0 | 0 | 0 | 0 | 1 | 1 | 0 | 0 | 1 | 1 |
| Comins, JD ^166^ | 2013 | Denmark | Anterior cruciate ligament deficiency reconstruction | Symptoms, activity limitations, and psychosocial consequences | Adults | 1 | 1 | KNEES – ACL | 0 | 1 | 1 | 0 | 1 | 0 | 1 | 0 | 1 | 2 |
| Dellon, ES ^167^ | 2013 | USA | Eosinophilic esophagitis | Dysphagia | Adolescents and adults | 1 | 5 | DSQ | 0 | 0 | 0 | 0 | 1 | 1 | 0 | 0 | 1 | 1 |
| DeWalt, DA ^168^ | 2013 | USA | Generic | Social health | Children | 2 | - | - | 2 | 1 | 0 | 0 | 1 | 1 | 0 | 0 | 1 | 3 |
| Doi, T ^169^ | 2013 | Japan | Osteoporotic back pain with vertebral fracture | Health status | Adults | 1 | 3 | JQ22 | 0 | 0 | 0 | 0 | 1 | 0 | 0 | 0 | 0 | 0 |
| Flynn, KE ^170^ | 2013 | USA | Cancer | Sexual Function and satisfaction | Adults | 1 | 2 | PROMIS® SexFS | 0 | 1 | 0 | 0 | 1 | 1 | 0 | 0 | 1 | 2 |
| Gabel, CP ^171^ | 2013 | Australia | Spine conditions | Functional status | Adults | 1 | 5 | SFI | 0 | 0 | 0 | 0 | 1 | 0 | 1 | 0 | 1 | 1 |
| Gordon, SE ^172^ | 2013 | New Zealand | Mental health | Recovery | Adults | 3 | 4 | - | 0 | 0 | 0 | 1 | 1 | 0 | 1 | 0 | 1 | 2 |
| Ridgeway, JL ^173^ | 2013 | USA | Diabetes | Quality of life | Adults | 1 | 3 | PROQOL | 0 | 0 | 0 | 1 | 1 | 0 | 0 | 0 | 1 | 1 |
| Kiltz, U ^174^ | 2013 | Germany | Ankylosing spondylitis | Function | Adults | 1 | 3 | ASAS HI | 0 | 0 | 0 | 1 | 1 | 0 | 0 | 0 | 1 | 1 |
| Lopez-Pousa, S ^175^ | 2013 | Spain | Fibromyalgia | Symptoms | Adults | 1 | 3 | CRSFS | 0 | 1 | 0 | 0 | 1 | 0 | 0 | 0 | 1 | 1 |
| Morley, D ^176^ | 2013 | England | A variety of health conditions | Participation and activities | Adults | 3 | 5 | Ox-PAQ | 0 | 0 | 1 | 0 | 1 | 1 | 1 | 0 | 1 | 2 |
| Pilkonis, PA ^177^ | 2013 | USA | Alcohol use | Negative and positive consequences of alcohol use, and negative and positive expectancies regarding drinking | Adults | 1 | 4 | - | 0 | 1 | 0 | 0 | 1 | 1 | 0 | 0 | 1 | 2 |
| Shembel, AC ^178^ | 2013 | USA | Chronic cough related to the upper airway | Symptoms | Adults | 1 | 3 | CSI | 0 | 0 | 0 | 0 | 1 | 0 | 0 | 0 | 0 | 0 |
| Thomson, HJ ^179^; Winters, ZE ^180^ | 2013 | England | Breast reconstruction | Health-related quality of life and satisfaction | Adults | 1 | 1 | BRR module | 0 | 0 | 1 | 0 | 1 | 1 | 0 | 0 | 1 | 2 |
| Welk, B ^181^ | 2013 | Canada | Neurogenic bladder dysfunction | Symptoms and bladder-related consequences | Adults | 1 | 5 | NBSS | 0 | 0 | 1 | 0 | 1 | 0 | 1 | 0 | 1 | 2 |
| Wilcox, TK ^182^ | 2013 | USA | Chronic obstructive pulmonary disease | Dyspnea | Adults | 1 | 3 | SOBDA | 2 | 1 | 0 | 0 | 1 | 1 | 0 | 0 | 1 | 3 |
| Augustin, M ^183^ | 2014 | Germany | Psoriasis and psoriatic arthritis | Quality of life | Adults | 1 | 3 | NAPPA-QoL | 0 | 0 | 0 | 1 | 1 | 0 | 0 | 0 | 1 | 1 |
| Augustin, M ^183^ | 2014 | Germany | Psoriasis and psoriatic arthritis | Treatment benefits | Adults | 1 | 3 | NAPPA-PBI | 0 | 0 | 0 | 1 | 1 | 0 | 0 | 0 | 1 | 1 |
| Augustin, M ^183^ | 2014 | Germany | Psoriasis and psoriatic arthritis | Severity | Adults | 1 | 3 | NAPPA-CLIN | 0 | 0 | 0 | 1 | 1 | 0 | 0 | 0 | 1 | 1 |
| Dawson, J ^184^ | 2014 | England | Total knee replacement | Activity and participation | Adults | 1 | 1 | OKS-APQ | 0 | 0 | 1 | 0 | 1 | 0 | 0 | 0 | 1 | 1 |
| Dean, K ^185^ | 2014 | England | Mild cognitive impairment | Quality of life | Adults | 1 | 3 | MCQ | 0 | 1 | 1 | 0 | 1 | 0 | 0 | 0 | 1 | 1 |
| Eberhart, NK ^186^ | 2014 | USA | Asthma | Quality of life | Adults | 1 | 3 | - | 2 | 1 | 0 | 0 | 1 | 1 | 0 | 0 | 1 | 3 |
| Naegeli, AN ^187^ | 2014 | USA | Osteoporosis | Physical function | Adults | 1 | 3 | OPAQ-PF | 0 | 0 | 1 | 0 | 1 | 1 | 0 | 0 | 1 | 2 |
| Reddy, P ^188^ | 2014 | England | Parkinson’s disease | Symptom severity and expectations of therapy | Adults | 1 | 3 | PRO-APD | 0 | 0 | 0 | 0 | 1 | 0 | 1 | 0 | 1 | 1 |
| Withers, KL ^189^ | 2014 | Wales | Cardiac ablation procedures for cardiac arrhythmias | Quality of life | Adults | 1 | 1 | - | 0 | 0 | 0 | 0 | 1 | 0 | 0 | 0 | 0 | 0 |

REFERENCE LIST

1. Meenan RF, Gertman PM, Mason JH. Measuring health status in arthritis. Arthritis and Rheumatism 1980; 23.

2. Hunt SM, McEwen JM. The development of a subjective health indicator. Sociology of Health and Illness 1980; 2: 232-246.

3. Neff EJ, Dale JC. Assessment of quality of life in school-aged children: a method--phase I. Maternal Child Nursing Journal 1990; 19: 313-320.

4. Corso DM, Pucino F, DeLeo JM, Calis KA, Gallelli JF. Development of a questionnaire for detecting potential adverse drug reactions. Annals of Pharmacotherapy 1992; 26: 890-896.

5. Ware Jr JE, Sherbourne CD. The MOS 36-item short-form health survey (SF-36). I. Conceptual framework and item selection. Medical care 1992; 30: 473-483.

6. Cella DF, Tulsky DS, Gray G, et al., The Functional Assessment of Cancer Therapy scale: development and validation of the general measure. Journal of Clinical Oncology 1993; 11: 570-579.

7. Garratt AM, Macdonald LM, Ruta DA, Russell IT, Buckingham JK, Krukowski ZH. Towards measurement of outcome for patients with varicose veins. Quality in Health Care 1993; 2: 5-10.

8. Levine DW, Simmons BP, Koris MJ, et al. A self-administered questionnaire for the assessment of severity of symptoms and functional status in carpal tunnel syndrome. The Journal of Bone & Joint Surgery 1993; 75: 1585-1592.

9. Harwood RH, Rogers A, Dickinson E, Ebrahim S. Measuring handicap: the London Handicap Scale, a new outcome measure for chronic disease. Quality in health care 1994; 3: 11-16.

10. Hays RD, Kallich JD, Mapes DL, Coons SJ, Carter WB. Development of the Kidney Disease Quality of Life (KDQOLTM) Instrument. Quality of Life Research 1994; 3: 329-338.

11. Ruta DA, Garratt AM, Wardlaw D, Russel IT. Developing a reliable and valid measure of health outcome for patients with low back pain. Spine Volume 1994; 19: 1887-1896.

12. Eisen SV, Dill DL, Grob MC. Reliability and Validity of a Brief Patient-Report Instrument for Psychiatric Outcome Evaluation. Hospital and Community Psychiatry 1994; 45: 242-247.

13. Barry MJ, Fowler Jr FJ, O'Leary MP, Bruskewitz RC, Holtgrewe HL, Mebust WK. Measuring disease-specific health status in men with benign prostatic hyperplasia. Measurement Committee of The American Urological Association. Medical care 1995; 33(4 Suppl): AS145-AS155.

14. Kolotkin R, Head S, Hamilton M, Tse CJ. Assessing impact of weight on quality of life. Obesity Research 1995; 3: 49-56.

15. Kopec JA, Esdaile JM, Abrahamovicz M, et al. The quebec back pain disability scale. Spine 1995; 20: 341-352.

16. Peto V, Jenkinson C, Fitzpatrick R, Greenhail R. The development and validation of a short measure of functioning and well being for individuals with Parkinson’s disease. Quality of Life Research 1995; 4: 241-248.

17. Daltroy LH, Cats-Bari WL, Katz JN, Fossel AH, Liang MH. The North American spine society lumbar spine outcome assessment instrument; Reliability and validity tests. SPINE Volume 1996; 21: 741-749.

18. Martin DP, Engelberg R, Agel J, Snapp D, Swiontkowski ME. Development of a Musculoskeletal Extremity Health Status Instrument: The Musculoskeletal Function Assessment Instrument. Journal of Orthopedic Research 1996; 14: 173-181.

19. Hudak PL, Amadia PC, Bombardier C, the upper extremity collaborative group. Development of an upper extremity outcome measure: The DASH (disabilities of the arm, shoulder, and head). American Journal of Industrial Medicine 1996; 29: 602-608.

20. Dawson J, Fitzpatrick R, Carr A, Murray D. Questionnaire on the perceptions of patients about total hip replacement. The Journal of Bone & Joint Surgery 1996; 78-B: 185-190.

21. Dawson J, Fitzpatrick R, Carr A, Murray D. Questionnaire on the perceptions of patients about shoulder surgery. The Journal of Bone & Joint Surgery 1996; 78: 593-600.

22. Lambert MJ, Burlingame GM, Umphress VH, et al., The Reliability and Validity of the Outcome Questionnaire. Clinical Psychology and Psychotherapy 1996; 3: 249-258.

23. Sprangers MA, Groenvold M, Arraras JI, et al., The European Organization for Research and Treatment of Cancer breast cancer-specific quality-of-life questionnaire module: first results from a three-country field study. Journal of Clinical Oncology 1996; 14: 2756-2768.

24. Stucki G, Daltroy L, Liang MH, Lipson SJ, Fossel AH, Katz JN. Measurement properties of a self-administered outcome measure in lumbar spinal stenosis. SPINE Volume 1996; 21: 796- 803.

25. Fletcher AE, Ellwein LB, Selvaraj SS, Vijaykumar VV, Rahmathullah R, Thulasiraj RD. Measurements of vision function and quality of life in patients with cataracts in southern india: Report of instrument development. Archives of Ophthalmology 1997; 115: 767-774.

26. The WHOQOL Group. The world health organization quality of life assessment (WHOQOL): Development and general psychometric properties. Social Science and Medicine 1998; 46: 1569-1585.

27. Irrgang JJ, Snyder-Mackler L, Wainner RS. Development of a Patient-Reported Measure of Function of the Knee. The Journal of Bone & Joint Surgery 1998; 80-A: 1132-1145.

28. Dawson J, Fitzpatrick R, Murray D, Carr A. Questionnaire on the perceptions of patients about total knee replacement. The Journal of Bone & Joint Surgery 1998; 80-B: 63-69.

29. Deyo RA, Battie M, Beurskens AJHM, et al. Outcome measures for low back pain research. Spine 1998; 23: 2003-2013.

30. Roos EM , Harald PR, Lohmander S, Ekdahl C, Beynnon BD. Knee Injury and Osteoarthritis Outcome Score: Development of a Self-Administered Outcome Measure. Journal of Orthopaedic and Sports Physical Therapy 1998; 78: 88-96.

31. Bradley C, Todd C, Gorton T, Symonds E, Martin A, Plowright R. The development of an individualized questionnaire measure of perceived impact of diabetes on quality of life: the ADDQoL. Quality of Life Research 1999; 8: 79-91.

32. Hawthorne G, Richardson J, Osborne R. The Assessment of Quality of Life (AQoL) instrument: a psychometric measure of Health-Related Quality of Life. Quality of Life Research 1999; 8: 209-224.

33. Haher TR, Gorup JM, Shin TM, et al. Results of the Scoliosis Research Society instrument for evaluation of surgical outcome in adolescent idiopathic scoliosis. A multicenter study of 244 patients. SPINE Volume 1999; 24: 1435 -1440.

34. Hearn J, Higginson IJ. Development and validation of a core outcome measure for palliative care: the palliative care outcome scale. Palliative Care Core Audit Project Advisory Group. Quality of Health Care 1999; 8: 219-227.

35. Varni JW, Seid M, Rode CA. The PedsQL™: Measurement Model for the Pediatric Quality of Life Inventory. Medical Care 1999; 37: 126-139.

36. Younossi ZM, Guyatt G, Kiwi M, Boparai N, King D. Development of a disease specific questionnaire to measure health related quality of life in patients with chronic liver disease. Gut 1999; 45: 295–300.

37. Garrow AP, Papageorgioua AC, Silmana AJ, Thomasa E, Jaysonb MYV, Macfarlanea GJ. Development and validation of a questionnaire to assess disabling foot pain. Pain 2000; 85: 107-113.

38. Cox R, Hyde H, Gatehouse S, et al., Optimal Outcome Measures, Research Priorities, and International Cooperation. Ear & Hearing 2000; 21: 106S-115S.

39. Evans C, Mellor-Clark J, Margison F, et al. CORE: Clinical Outcomes in Routine Evaluation. Journal of Mental Health Policy and Economics, 2000.

40. Firth H, Grimes A, Poppleton H, Hall R, Richold P. Assessment of parents' concerns and evaluation of outcomes. Journal of Public Health Medicine 2000; 22: 473-478.

41. Vitale S, Schein OD, Meinert CL, Steinberg EP. The Refractive Status and Vision Profile A Questionnaire to Measure Vision-related Quality of Life in Persons with Refractive Error. Ophthalmology 2000; 107: 1529–1539.

42. Hobart J, Lamping D, Fitzpatrick R, Riazi A, Thompson A. The multiple sclerosis impact scale (MSIS-29): a new patient-based outcome measure. Brain 2001; 124: 962-973.

43. Morgan MB, Crayford T, Murrin B, Fraser SC. Developing the Vascular Quality of Life Questionnaire: a new disease-specific quality of life measure for use in lower limb ischemia. Journal of Vascular Surgery 2001; 33: 679-687.

44. Rabin R, De Charro F. EQ-SD: a measure of health status from the EuroQol Group. Annals of Medicine 2001; 33: 337-343.

45. Vickery CW, Blazeby JM, Conroy T, et al. Development of an EORTC disease-specific quality of life module for use in patients with gastric cancer. European Journal of Cancer 2001; 37: 966-971.

46. Bialocerkowski AE. Difficulties associated with wrist disorders--a qualitative study. Clinical Rehabilitation 2002; 16: 429 -440.

47. Bialocerkowski AE, Grimmer KA, Bain GI. Development of a patient-focused wrist outcome instrument. Hand Clinics 2003; 19: 437-448, ix.

48. Wilson JTL, Edwards P, Fiddes H, Stewart E, Teasdale GM. Reliability of Postal Questionnaires for the Glasgow Outcome Scale. Journal of Neurotrauma 2002; 19: 999-1005.

49. Spies JB, Coyne K, Guaou Guaou N, Boyle D, Skyrnarz-Murphy K, Gonzalves SM. The UFS-QOL, a New Disease-Specific Symptom and Health-Related Quality of Life Questionnaire for Leiomyomata. Obstetrics and Gynecology 2002; 99: 290–300.

50. Kazis LE, Liang MH, Lee A. et al., The development, validation, and testing of a health outcomes burn questionnaire for infants and children 5 years of age and younger: American Burn Association/Shriners Hospitals for Children. Journal of Burn Care Rehabilitation 2002; 23: 196-207.

51. Lamping DL, Schroter S, Marquis P, Marrel A, Duprat-Lomon I, Sagnier PP. The community-acquired pneumonia symptom questionnaire: A new, patient-based outcome measure to evaluate symptoms in patients with community-acquired pneumonia. Chest 2002; 122: 920-929.

52. Manterola C, Munoz S, Grande L, Bustos L. Initial validation of a questionnaire for detecting gastroesophageal reflux disease in epidemiological settings. Journal of Clinical Epidemiology 2002; 55: 1041-1045.

53. Thompson DR, Jenkinson C, Roebuck A, Lewin RJ, Boyle RM, Chandola T. Development and validation of a short measure of health status for individuals with acute myocardial infarction: the myocardial infarction dimensional assessment scale (MIDAS). Quality of Life Research 2002; 11: 535-543.

54. Kosinski M, Bayliss MS, Bjorner JB, et al. A six-item short-form survey for measuring headache impact: The HIT-6. Quality of Life Research 2003; 12: 963–974.

55. Macran S, Kind P, Collingwood J, Hull R, McDonald I, Parkinson L. Evaluating Podiatry Services: Testing a Treatment Specific Measure of Health Status. Quality of Life Research 2003; 12: 177-188.

56. Arranz P, Remor E, Quintana M, et al. Development of a new disease-specific quality-of-life questionnaire to adults living with haemophilia. Haemophilia 2004; 10: 376-382.

57. Chasan-Taber L, Schmidt MD, Roberts DE, Hosmer D, Markenson G, Freedson PS. Development and Validation of a Pregnancy Physical Activity Questionnaire. Medicine & Science in Sports & Exercise 2004; 36: 1750-1760.

58. McMillan CV, Bradley C, Woodcock A, Razvi S Weaver JU. Design of New Questionnaires to Measure Quality of Life and Treatment Satisfaction in Hypothyroidism. Thyroid 2004; 14.

59. Haley SM, Coster WJ, Andres PL, et al., Activity outcome measurement for postacute care. Medical Care 2004; 42(1 Suppl): I49-I61.

60. Mystakidou K, Tsilika E, Kouloulias V, et al. The "Palliative Care Quality of Life Instrument (PQLI)" in terminal cancer patients. Health anf Quality of Life Outcomes 2004; 2: 8.

61. McKenna SP, Doward LC, Whalley D, Tennant A, Emery P, Veale DJ. Development of the PsAQoL: a quality of life instrument specific to psoriatic arthritis. Annals of the Rheumatic Diseases 2004; 63: 162-169.

62. Young NL, Bradley CS, Blanchette V, et al. Development of a health-related quality of life measure for boys with haemophilia: the Canadian Haemophilia Outcomes – Kids Life Assessment Tool (CHO-KLAT). Haemophilia 2004; 10: 34-43.

63. Spertus J, Jones P, Poler S, Rocha-Singh K. The peripheral artery questionnaire: a new disease-specific health status measure for patients with peripheral arterial disease. American Heart Journal 2004; 147: 301-308.

64. Van Genderen FR, Van Meeteren NL, Van der Bom JG, et al. Functional consequences of haemophilia in adults: the development of the Haemophilia Activities List. Haemophilia 2004; 10: 565-571.

65. Becker S, Kruse A, Schröder J, Seidl U. Das Heidelberger Instrument zur Erfassung von Lebensqualität bei Demenz (H.I. L.DE.). Zeitschrift für Gerontologie und Geriatrie 2005; 38: 108-121.

66. Chen CC, Granger CV, Peimer CA, Moy OJ, Wald, S. Manual Ability Measure (MAM-16): a preliminary report on a new patient-centred and task-oriented outcome measure of hand function. The Journal of Hand Surgery British 2005; 30: 207-216.

67. Malaty HM, Abudayyeh S, O'Malley KJ, et al. Development of a multidimensional measure for recurrent abdominal pain in children: population-based studies in three settings. Pediatrics 2005; 115: e210-e215.

68. Rat AC, Coste J, Pouchot J, et al. OAKHQOL: a new instrument to measure quality of life in knee and hip osteoarthritis. Journal of Clinical Epidemiology 2005; 58: 47-55.

69. Smith SC, Lamping DL, Banerjee S, et al. Measurement of health-related quality of life for people with dementia: development of a new instrument (DEMQOL) and an evaluation of current methodology. Health Technology Assessment 2005; 9: 1-93.

70. Vidhubala E, Kannan RR, Mani SC, et al. Validation of quality of life questionnaire for patients with cancer – Indian scenario. Indian Journal of Cancer 2005; 42: 138-144.

71. Schaefer AM, Phoenix C, Elson JL, McFarland R, Chinnery PF, Turnbull DM. Mitochondrial disease in adults: A scale to monitor progression and treatment. Neurology 2006; 66: 1932–1934.

72. Atkinson MJ, Lohs J, Kuhagen I, Kaufman J, Bhaidani S. A promising method for identifying cross-cultural differences in patient perspective: the use of Internet-based focus groups for content validation of new patient reported outcome assessments. Health and Quality of Life Outcomes 2006; 4: 64.

73. Cano SJ, Browne JP, Lamping DL, Roberts AHN, McGrouther DA, Black NA. The Patient Outcomes of Surgery-Head/Neck (POS-Head/Neck): A new patient-based outcome measure. Journal of Plastic, Reconstructive and Aesthetic Surgery 2006; 59: 65-73.

74. Chen TY, Landmann MG, Potter JC, van Rij AM. Questionnaire to Aid Priority and Outcomes Assessment in Gallstone Disease. ANZ Journal of Surgery 2006; 76: 569-574.

75. Urbach DR, Harnish JL, McIlroy JH, Streiner DL. A Measure of Quality of Life after Abdominal Surgery. Quality of Life Research 2006; 15: 1053-1061.

76. Colwell HH, Hunt BJ, Pasta DJ, Palo WA, Mathias SD, Joseph-Ridge N. Gout Assessment Questionnaire: initial results of reliability, validity and responsiveness. International Journal of Clinical Practice 2006; 60: 1210–1217.

77. McKenna SP, Doughty N, Meads DM, Doward LC, Pepke-Zaba J. The Cambridge Pulmonary Hypertension Outcome Review (CAMPHOR): a measure of health-related quality of life and quality of life for patients with pulmonary hypertension. Quality of Life Research 2006; 15: 103-115.

78. McMillan CV, Bradley C, Gibney J, Russell-Jones DL, Sonksen PH. Preliminary development of the new individualized HDQoL questionnaire measuring quality of life in adult hypopituitarism. Journal of Evaluation in Clinical Practice 2006; 12: 501-514.

79. Taback NA, Bradley C. Validation of the Genital Herpes Treatment Satisfaction Questionnaire (GHerpTSQ) in Status and Change Versions. Quality of Life Research 2006; 15: 1043-1052.

80. Nordyke RJ, Chang CH, Chiou CF, Wallace JF, Yao B, Schwartzberg LS. Validation of a patient satisfaction questionnaire for anemia treatment, the PSQ-An. Health and Quality of Life Outcomes 2006; 4: 28.

81. Osborne RH, Hawkins M, Sprangers MA. Change of perspective: a measurable and desired outcome of chronic disease self-management intervention programs that violates the premise of preintervention/postintervention assessment. Arthritis & Rheumatism 2006; 55: 458-465.

82. Pollak E, Mühlan H, Von Mackensen S, Bullinger M, Haemo-Qol, Group. The Haemo-QoL Index: developing a short measure for health-related quality of life assessment in children and adolescents with haemophilia. Haemophilia 2006; 12: 384-392.

83. Schrag A, Selai C, Quinn N, et al. Measuring quality of life in PSP: The PSP-QoL. Neurology 2006; 67: 39-44.

84. Vasiliadis E, Grivas TB, Gkoltsiou K. Development and preliminary validation of Brace Questionnaire (BrQ): a new instrument for measuring quality of life of brace treated scoliotics. Scoliosis 2006; 1: 7.

85. Yaruss JS, Quesal RW. Overall Assessment of the Speaker's Experience of Stuttering (OASES): documenting multiple outcomes in stuttering treatment. Journal of Fluency Disorders 2006; 31: 90-115.

86. Fukui M, Chiba K, Kawakami M, et al. An outcome measure for patients with cervical myelopathy: Japanese Orthopaedic Association Cervical Myelopathy Evaluation Questionnaire (JOACMEQ): Part 1. Journal of Orthopaedic Science 2007; 12: 227-240.

87. Gandek B, Sinclair SJ, Jette AM, Ware JE Jr. Development and initial psychometric evaluation of the participation measure for post-acute care (PM-PAC). American Journal of Physical Medicine and Rehabilitation 2007; 86: 57-71.

88. Nilsson-Helander K, Thomeé R, Silbernagel KG, et al. The Achilles tendon Total Rupture Score (ATRS): development and validation. American Journal of Sports Medicine 2007; 35: 421-426.

89. Osborne RH, Elsworth GR, Whitfield K. The Health Education Impact Questionnaire (heiQ): an outcomes and evaluation measure for patient education and self-management interventions for people with chronic conditions. Patient Education and Counseling 2007; 66: 192-201.

90. Rodrigues G, Bauman G, Lock M, D'Souza D, Mahon J. Psychometric properties of a prostate cancer radiation late toxicity questionnaire. Health and Quality of Life Outcomes 2007; 5: 29.

91. Schrag A, Selai C, Mathias C, et al. Measuring health-related quality of life in MSA: The MSA-QoL. Movement Disorders 2007; 22: 2332-2338.

92. Shirado O, Doi T, Akai M, Fujino K, Hoshino Y, Iwaya T. An outcome measure for Japanese people with chronic low back pain: an introduction and validation study of Japan Low Back Pain Evaluation Questionnaire. Spine 2007; 32: 3052-3059.

93. Walter OB, Becker J, Bjorner JB, Fliege H, Klapp BF, Rose M. Development and evaluation of a computer adaptive test for 'Anxiety' (Anxiety-CAT). Quality of Life Research 2007; 16 Suppl 1: 143-155.

94. Arbuckle R, Atkinson MJ, Clarck M, et al. Patient experiences with oily skin: the qualitative development of content for two new patient reported outcome questionnaires. Health and Quality of Life Outcomes 2008; 6: 80.

95. Bouffioulx E, Arnould C, Thonnard J. Satis-stroke: A satisfaction measure of activities and participation in the actual environment experienced by patients with chronic stroke. Journal of Rehabilitation Medicine 2008; 40: 836-843.

96. Caty GD, Arnould C, Stoquart GG, Thonnard JL, Lejeune TM. ABILOCO: a Rasch-built 13-item questionnaire to assess locomotion ability in stroke patients. Archives of Physical Medicine & Rehabilitation 2008; 89: 284-290.

97. Caty GD, Arnould C, Thonnard JL, Lejeune TM. ABILOCO-Kids: a Rasch-built 10-item questionnaire for assessing locomotion ability in children with cerebral palsy. Journal of Rehabilitation & Medicine 2008; 40: 823-830.

98. Dawson JH, Doll I, Boller R, et al. The development and validation of a patient reported questionnaire to assess outcomes of elbow surgery. The Journal of Bone & Joint Surgery 2008; 90: 466-473.

99. Deering DE, Sellman JD, Adamson SJ, Horn J, Frampton CM. Development of a brief treatment instrument for routine clinical use with methadone maintenance treatment clients: the methadone treatment index. Substance Use & Misuse 2008; 43: 1666-1680.

100. Kushner JA, Lawrence HP, Shoval I, et al. Development and validation of a Patient-Reported Oral Mucositis Symptom (PROMS) scale. Journal (Canadian Dental Association) 2008; 74: 59.

101. McMillan C, Bradley C, Razvi S, Weaver J. Evaluation of new measures of the impact of hypothyroidism on quality of life and symptoms: the ThyDQoL and ThySRQ. Value in Health 2008; 11: 285-294.

102. Rentz A, Flood E, Altisent C, et al. Cross-cultural development and psychometric evaluation of a patient-reported health-related quality of life questionnaire for adults with haemophilia. Haemophilia 2008; 14: 1023-1034.

103. Rose M, Bjorner JB, Becker J, Fries JF, Ware JE. Evaluation of a preliminary physical function item bank supported the expected advantages of the Patient-Reported Outcomes Measurement Information System (PROMIS). Journal of Clinical Epidemiology 2008; 61: 17-33.

104. Rosen R, Catania J, Lue T, et al. Impact of Peyronie's disease on sexual and psychosocial functioning: Qualitative findings in patients and controls. Journal of Sexual Medicine 2008; 5: 1977-1984.

105. Webster KE, Feller JA, Lambros C. Development and preliminary validation of a scale to measure the psychological impact of returning to sport following anterior cruciate ligament reconstruction surgery. Physical Therapy in Sport 2008; 9: 9-15.

106. Allvin R, Ehnfors M, Rawal N, Svensson E, Idvall E. Development of a questionnaire to measure patient-reported postoperative recovery: content validity and intra-patient reliability. Journal of Evaluation in Clinical Practice 2009; 15: 411-419.

107. Baumann I, Cerman Z, Sertel S, Skevas T, Klingmann C, Plinkert PK. Entwicklung und Validierung des Parotidectomy Outcome Inventory 8 (POI-8). Hno 2009; 57: 884-888.

108. Bauman I, Kurpiers B, Plinkert P, Praetorius M. Entwicklung und Validierung des Chronic Otitis Media Outcome Test 15 (COMOT-15). Hno 2009; 57: 889-895.

109. Blome C, Augustin M, Siepmann D, Phan NQ, Rustenbach SJ, Stander S. Measuring patient-relevant benefits in pruritus treatment: development and validation of a specific outcomes tool. British Journal of Dermatology 2009; 161: 1143-1148.

110. Blome C, Maares J, Diepgen T, Jeffrustenbach S, Augustin M. Measurement of patient-relevant benefits in the treatment of chronic hand eczema--a novel approach. Contact Dermatitis 2009; 61: 39-45.

111. Brod M, Hammer M, Christensen T, Lessard S, Bushnell DM. Understanding and assessing the impact of treatment in diabetes: the Treatment-Related Impact Measures for Diabetes and Devices (TRIM-Diabetes and TRIM-Diabetes Device). Health and Quality of Life Outcomes 2009; 7: 83.

112. Doward LC, McKenna SP, Meads DM, Twiss J, Eckert BJ. The development of patient-reported outcome indices for multiple sclerosis (PRIMUS). Multiple Sclerosis 2009; 15: 1092-1102.

113. Helbostad JL, Hølen JC, Jordhøy MS, et al. A first step in the development of an international self-report instrument for physical functioning in palliative cancer care: a systematic literature review and an expert opinion evaluation study. Journal of Pain and Symptom Management 2009; 37: 196-205.

114. Jette AM, McDonough CM, Haley SM, et al. A computer-adaptive disability instrument for lower extremity osteoarthritis research demonstrated promising breadth, precision, and reliability. Journal of Clinical Epidemiology 2009. 62: 807-815.

115. Katz JN, Wright EA, Baron JA, Losina E. Development and validation of an index of musculoskeletal functional limitations. BMC Musculoskeletal Disorders 2009; 10: 62.

116. Li T, Wells G, Westhovens R, Tugwell P. Validation of a simple activity participation measure for rheumatoid arthritis clinical trials. Rheumatology (Oxford) 2009; 48: 170-5.

117. Pollard B, Dixon D, Dieppe P, Johnston M. Measuring the ICF components of impairment, activity limitation and participation restriction: an item analysis using classical test theory and item response theory. Health and Quality of Life Outcomes 2009; 7: 41.

118. Pusic AL, Klassen AF, Scott AM, Klok JA, Cordeiro PG, Cano SJ. Development of a new patient-reported outcome measure for breast surgery: The BREAST-Q. Plastic and Reconstructive Surgery 2009; 124: 345-353.

119. Pusic AL, Reavey PL, Klassen AF, Scott A, McCarthy C, Cano SJ. Measuring Patient Outcomes in Breast Augmentation: Introducing the BREAST-Q(copyright) Augmentation Module. Clinics in Plastic Surgery 2009; 36: 23-32.

120. Shaikh N, Hoberman A, Paradise JL, et al. Development and preliminary evaluation of a parent-reported outcome instrument for clinical trials in acute otitis media. The Pediatric Infectious Disorders Journal 2009; 28: 5-8.

121. Agarwal R. Developing a self-administered CKD symptom assessment instrument. Nephrology Dialysis Transplantation 2010; 25: 160-166.

122. Aghayev E, Sprott H, Bohler D, Roder C, Muller U. Sleep quality, the neglected outcome variable in clinical studies focusing on locomotor system; a construct validation study. BMC Musculoskeletal Disorders 2010; 11: 224.

123. Arbuckle R, Abetz L, Durmer JS, et al. Development of the Pediatric Restless Legs Syndrome Severity Scale (P-RLS-SS): a patient-reported outcome measure of pediatric RLS symptoms and impact. Sleep Medicine 2010; 11: 897-906.

124. Bishop FL, Barlow F, Walker J, McDermott C, Lewith GT. The development and validation of an outcome measure for spiritual healing: A mixed methods study. Psychotherapy and Psychosomatics 2010; 79: 350-362.

125. Bode RK, Hahn EA, DeVellis R, Cella D. Measuring Participation: The Patient-Reported Outcomes Measurement Information System Experience. Archives of Physical Medicine and Rehabilitation 2010; 91: S60-S65.

126. Deal LS, DiBenedetti DB, Williams VS, Fehnel SE. The development and validation of the daily electronic Endometriosis Pain and Bleeding Diary. Health and Quality of Life Outcomes 2010; 8: 64.

127. Deal LS, DiBenedetti DB, Williams VS, Fehnel SE. Development and psychometric evaluation of the Endometriosis Treatment Satisfaction Questionnaire. Quality of Life Research 2010; 19: 899-905.

128. El Miedany Y, El Gaafary M, Youssef SS, Palmer D. Incorporating patient reported outcome measures in clinical practice: Development and validation of a proms questionnaire for inflammatory arthritis. Rheumatology 2010; 49: i163-il64.

129. El Miedany Y, El Gaafary M, Youssef SS, Palmer D. Towards a multidimensional patient reported outcome measures assessment: development and validation of a questionnaire for patients with ankylosing spondylitis/spondyloarthritis. Joint Bone Spine 2010; 77: 575-581.

130. Haywood KL, Garratt AM, Jordan KP, Healey EL, Packham JC. Evaluation of ankylosing spondylitis quality of life (EASi-QoL): Reliability and validity of a new patient-reported outcome measure. Journal of Rheumatology 2010; 37: 2100- 2109.

131. Herlyn K, Hellmich B, Seo P, Merkel PA. Patient-reported outcome assessment in vasculitis may provide important data and a unique perspective. Arthritis Care & Research 2010; 62: 1639-1645.

132. Klassen AF, Cano SJ, Scott A, Snell L, Pusic AL. Measuring patient-reported outcomes in facial aesthetic patients: development of the FACE-Q. Facial Plastic Surgery 2010; 26: 303-309.

133. Leidy NK, Wilcox TK, Jones PW, et al. Development of the EXAcerbations of Chronic Obstructive Pulmonary Disease Tool (EXACT): a patient-reported outcome (PRO) measure. Value in Health 2010; 13: 965-975.

134. Nicklin J, Cramp F, Kirwan J, Urban M, Hewlett S. Collaboration with patients in the design of patient-reported outcome measures: capturing the experience of fatigue in rheumatoid arthritis. Arthritis Care & Research 2010; 62: 1552-1558.

135. Von Mackensen S, Czepa D, Herbsleb M, Hilberg T. Development and validation of a new questionnaire for the assessment of subjective physical performance in adult patients with haemophilia--the HEP-Test-Q. Haemophilia 2010; 16: 170-178.

136. Von Steinbüchel N, Wilson L, Gibbons H, et al. Quality of Life after Brain Injury (QOLIBRI): scale development and metric properties. Journal of Neurotrauma 2010; 27: 1167-1185.

137. Zimmerman M, Chelminski I, Young D, Dalrymple K. A clinically useful anxiety outcome scale. Journal of Clinical Psychiatry 2010; 71: 534-542.

138. Gibbons CJ, Mills RJ, Thornton EW, et al. Development of a patient reported outcome measure for fatigue in Motor Neurone Disease: The Neurological Fatigue Index (NFI-MND). Health and Quality of Life Outcomes 2011: 101.

139. Jackson MJ, Sciberras J, Mangera A, et al. Defining a patient-reported outcome measure for urethral stricture surgery. European Urology 2011; 60: 60-68.

140. Matza LS, Phillips GA, Revicki DA, Murray L, Malley KG. Development and validation of a patient-report measure of fatigue associated with depression. Journal of Affective Disorders 2011; 134: 294-303.

141. McAllister M, Dunn G, Todd C. Empowerment: qualitative underpinning of a new clinical genetics-specific patient-reported outcome. European Journal of Human Genetics 2011; 19: 125-130.

142. McAllister M, Wood AM, Dunn G, Shiloh S, Todd C. The Genetic Counseling Outcome Scale: a new patient-reported outcome measure for clinical genetics services. Clinical Genetics 2011; 79: 413-424.

143. Ritenbaugh C, Nichter M, Nichter MA, et al. Developing a patient-centered outcome measure for complementary and alternative medicine therapies I: defining content and format. BMC Complementary Alternative Medicine 2011; 11: 135.

144. Thompson JJ, Kelly KL, Ritenbaugh C, Hopkins AL, Sims CM, Coons SJ. Developing a patient-centered outcome measure for complementary and alternative medicine therapies II: refining content validity through cognitive interviews. BMC Complementary Alternative Medicine 2011; 11: 136.

145. Amtmann D, Bamer AM, Cook KF, Askew RL, Noonan VK, Brockway JA. University of Washington self-efficacy scale: a new self-efficacy scale for people with disabilities. Archives of Physical Medicine & Rehabilitation 2012; 93: 1757-1765.

146. Drapalski AL, Medoff D, Unick GJ, Velligan DI, Dixon LB, Bellack AS. Assessing Recovery of People With Serious Mental Illness: Development of a New Scale. Psychiatric Services 2012; 63: 48–53.

147. Aufwerber S, Hagstromer M, Heijne A. Donor-site-related functional problems following anterior cruciate ligament reconstruction: development of a self-administered questionnaire. Knee Surgery, Sports Traumatology, Arthroscopy 2012; 20: 1611-1621.

148. Chen RQ, Wong CM, Lam TH. Construction of a traditional Chinese medicine syndrome-specific outcome measure: the Kidney Deficiency Syndrome questionnaire (KDSQ). BMC Complementary Alternative Medicine 2012; 12.

149. Gabel CP, Melloh M, Burkett B, Michener LA. Lower limb functional index: development and clinimetric properties. Physical Therapy 2012; 92: 98-110.

150. Govender R, Lee MT, Davies TC, et al. Development and preliminary validation of a patient-reported outcome measure for swallowing after total laryngectomy (SOAL questionnaire). Clinical Otolaryngology 2012; 37: 452- 459.

151. Hocaoglu MB, Gaffan EA, Ho AK. The Huntington's Disease health-related Quality of Life questionnaire (HDQoL): a disease-specific measure of health-related quality of life. Clinical Genetics 2012; 81: 117-122.

152. Jolly M, Pickard AS, Block JA, et al. Disease-specific patient reported outcome tools for systemic lupus erythematosus. Seminars in Arthritis and Rheumatism 2012; 42: 56-65.

153. Kaufmann H, Malamut R, Norcliffe-Kaufmann L, Rosa K, Freeman R. The Orthostatic Hypotension Questionnaire (OHQ): validation of a novel symptom assessment scale. Clinical Autonomic Research 2012; 22: 79-90.

154. Lasch KE, Hassan M, Endicott J, et al. Development and content validity of a patient reported outcomes measure to assess symptoms of major depressive disorder. BMC Psychiatry 2012; 12: 34.

155. Mohtadi NG, Griffin DR, Pedersen ME, et al. The development and validation of a self-administered quality-of-life outcome measure for young, active patients with symptomatic hip disease: The International Hip Outcome Tool (iHOT-33). Arthroscopy 2012; 28: 595-610.

156. Mojtabai R, Corey-Lisle PK, Ip EH, et al. The Patient Assessment Questionnaire: initial validation of a measure of treatment effectiveness for patients with schizophrenia and schizoaffective disorder. Psychiatry Research 2012; 200: 857-866.

157. Otter SJ, Lucas K, Springett K, et al. Identifying patient-reported outcomes in rheumatoid arthritis: the impact of foot symptoms on self-perceived quality of life. Musculoskeletal Care 2012; 10: 65-75.

158. Pinder B, Lloyd AJ, Elwick H, Denys P, Marley J, Bonniaud V. Development and psychometric validation of the intermittent self-catheterization questionnaire. Clinical Therapy 2012; 34: 2302-2313.

159. Schmidutz F, Beirer M, Braunstein V, Bogner V, Wiedemann E, Biberthaler P. The Munich Shoulder Questionnaire (MSQ): development and validation of an effective patient-reported tool for outcome measurement and patient safety in shoulder surgery. Patient Safety in Surgery 2012; 6.

160. Skevas T, Klingmann C, Plinkert PK, Baumann I. [Development and validation of the Tonsillectomy Outcome Inventory 14]. HNO 2012; 60: 801-806.

161. Wagner LI, Robinson D Jr, Weiss M, et al. Content development for the Functional Assessment of Cancer Therapy-Multiple Myeloma (FACT-MM): use of qualitative and quantitative methods for scale construction. Journal of Pain and Symptom Management 2012; 43: 1094-1104.

162. Glaser AW, Fraser LK, Corner J, et al. Patient-reported outcomes of cancer survivors in England 1–5 years after diagnosis: a cross-sectional survey. BMJ Open 2013; 3.

163. Hareendran A, Palsgrove AC, Mocarski M, et al. The development of a patient-reported outcome measure for assessing nighttime symptoms of chronic obstructive pulmonary disease. Health and Quality of Life Outcomes 2013; 11: 104.

164. Bankstahl US, Gortelmeyer R. Measuring subjective complaints of attention and performance failures - development and psychometric validation in tinnitus of the self-assessment scale APSA. Health and Quality of Life Outcomes 2013; 11.

165. Gorecki C, Brown JM, Cano S, et al. Development and validation of a new patient-reported outcome measure for patients with pressure ulcers: the PU-QOL instrument. Health and Quality of Life Outcomes 2013; 11: 95.

166. Comins JD, Krogsgaard MR, Brodersen J. Development of the Knee Numeric-Entity Evaluation Score (KNEES-ACL): a condition-specific questionnaire. Scandinavian Journal of Medicine & Science in Sports 2013; 23: e293-e301.

167. Dellon ES, Irani AM, Hill MR, Hirano I. Development and field testing of a novel patient reported outcome measure of dysphagia in patients with eosinophilic esophagitis: The dysphagia symptom questionnaire. Gastroenterology 2013; 144: S487- S488.

168. Dewalt DA, Thissen D, Stucky BD, et al. PROMIS Pediatric Peer Relationships Scale: development of a peer relationships item bank as part of social health measurement. Health Psychology 2013; 32: 1093-1103.

169. Doi T, Akai M, Endo N, Fujino K, Iwaya T. Dynamic change and influence of osteoporotic back pain with vertebral fracture on related activities and social participation: Evaluating reliability and validity of a newly developed outcome measure. Journal of Bone & Mineral Metabolism 2013; 31: 663-673.

170. Flynn KE, Lin L, Cyranowski JM, et al. Development of the NIH PROMIS (R) Sexual Function and Satisfaction measures in patients with cancer. The Journal of Sexual Medicine 2013; 10 Suppl 1: 43-52.

171. Gabel CP, Melloh M, Burkett B, Michener LA. The Spine Functional Index: development and clinimetric validation of a new whole-spine functional outcome measure. Spine J 2013.

172. Gordon SE, Ellis PM, Siegert RJ, Walkey FH. Development of a self-assessed consumer recovery outcome measure: my voice, my life. Administration and Policy in Mental Health 2013; 40: 199-210.

173. Ridgeway JL, Beebe TJ, Chute CG, et al. A Brief Patient-Reported Outcomes Quality of Life (PROQOL) Instrument to Improve Patient Care. PLOS Medicine 2013; 10.

174. Kiltz U, van der Heijde D, Boonen A, et al. Development of a health index in patients with ankylosing spondylitis (ASAS HI)-final result of a global initiative based on the ICF guided by ASAS. Annals of Rheumatic disorders 2013; 72.

175. López-Pousa S, Garre-Olmo J, de Gracia M, Ribot J, Calvo-Perxas, L, Vilalta-Franch J. Development of a multidimensional measure of fibromyalgia symptomatology: The comprehensive rating scale for fibromyalgia symptomatology. Journal of Psychosomatic Research 2013.

176. Morley D, Dummett S, Kelly L, Dawson, J, Fitzpatrick R, Jenkinson C. The Oxford Participation and Activities Questionnaire: study protocol. Patient Related Outcome Measures 2013; 5: 1-6.

177. Pilkonis PA, Yu L, Colditz J, et al. Item banks for alcohol use from the Patient-Reported Outcomes Measurement Information System (PROMIS): use, consequences, and expectancies. Drug and Alcohol Dependence 2013; 130: 167-177.

178. Shembel AC, Rosen CA, Zullo TG, Gartner-Schmidt JL. Development and validation of the cough severity index: a severity index for chronic cough related to the upper airway. Laryngoscope 2013; 123: 1931-1936.

179. Thomson HJ, Winters ZE, Brandberg Y, Didier F, Blazeby JM, Mills J. The early development phases of a European Organisation for Research and Treatment of Cancer (EORTC) module to assess patient reported outcomes (PROs) in women undergoing breast reconstruction. European Journal of Cancer 2013; 49: 1018-1026.

180. Winters ZE, Balta V, Thomson HJ, et al. Phase III development of the European Organization for Research and Treatment of Cancer Quality of Life Questionnaire module for women undergoing breast reconstruction. British Journal of Surgery 2014; 101: 371-382.

181. Welk B, Morrow SA, Madarasz W, Potter P, Sequeira K. The conceptualization and development of a patient-reported neurogenic bladder symptom score. Journal of Research and Reports in Urology 2013; 5: 129-137.

182. Wilcox TK, Chen WH, Howard KA, et al. Item selection, reliability and validity of the Shortness of Breath with Daily Activities (SOBDA) questionnaire: A new outcome measure for evaluating dyspnea in chronic obstructive pulmonary disease. Health and Quality of Life Outcomes 2013; 11.

183. Augustin M, Blome C, Costanzo A, et al. Nail assessment in psoriasis and psoriatic arthritis (NAPPA): Development and validation of a tool for assessment of nail psoriasis outcomes. British Journal of Dermatology 2014; 170: 591-598.

184. Dawson J, Beard DJ, McKibbin H, Harris K, Jenkinson C, Price AJ. Development of a patient-reported outcome measure of activity and participation (the OKSAPQ) to supplement the Oxford knee score. The Bone & Joint Journal 2014; 96 B: 332-338.

185. Dean K, Jenkinson C, Wilcock G, Walker Z. The development and validation of a patient-reported quality of life measure for people with mild cognitive impairment. International Psychogeriatrics 2014; 26: 487-97.

186. Eberhart NK, Sherbourne CD, Edelen MO, Stucky BD, Sin NL, Lara M. Development of a measure of asthma-specific quality of life among adults. Quality of Life Research 2014; 23: 837-848.

187. Naegeli AN, Nixon A, Burge R, Gold DT, Silverman S. Development of the Osteoporosis Assessment Questionnaire--physical Function (OPAQ-PF): an osteoporosis-targeted, patient-reported outcomes (PRO) measure of physical function. Osteoporosis International 2014; 25: 579-588.

188. Reddy P, Martinez-Martin P, Brown RG, et al. Perceptions of symptoms and expectations of advanced therapy for Parkinson’s disease: preliminary report of a Patient-Reported Outcome tool for Advanced Parkinson’s disease (PRO-APD). Health and Quality of Life Outcomes 2014; 12: 11.

189. Withers KL, White J, Carolan-Rees G, et al. Patient reported outcome measures for cardiac ablation procedures: a multicentre pilot to develop a new questionnaire. Europace 2014.
